# Chemoselective and one-pot synthesis of novel coumarin-based cyclopenta[*c*]pyrans *via* base-mediated reaction of α,β-unsaturated coumarins and β-ketodinitriles†

**DOI:** 10.1039/d2ra00594h

**Published:** 2022-03-04

**Authors:** Behnaz Farajpour, Abdolali Alizadeh

**Affiliations:** Department of Chemistry, Tarbiat Modares University P. O. Box 14115-175 Tehran Iran

## Abstract

In this paper, the base-mediated cascade reactions of 4-chloro-3-vinyl coumarins with β-ketodinitriles were demonstrated, allowing the efficient synthesis of coumarin-based cyclopenta[*c*]pyran-7-carbonitriles with interesting chemoselectivity. These transformations include the domino-style formation of C–C/C–C/C–O bonds through a base-mediated nucleophilic substitution, Michael addition, tautomerization, *O*-cyclization, elimination, and aromatization. The presented synthetic strategy has many advantages such as simple and readily available starting materials, green solvent, highly chemoselective route, synthetically useful yields, and easy purification of products by washing them with EtOH (96%), described as GAP (Group-Assistant-Purification) chemistry.

## Abstract 2

In this paper, the base-mediated cascade reactions of 4-chloro-3-vinyl coumarins with β-ketodinitriles were demonstrated, allowing the efficient synthesis of coumarin-based cyclopenta[*c*]pyran-7-carbonitriles with interesting chemoselectivity.

## Graphical abstract

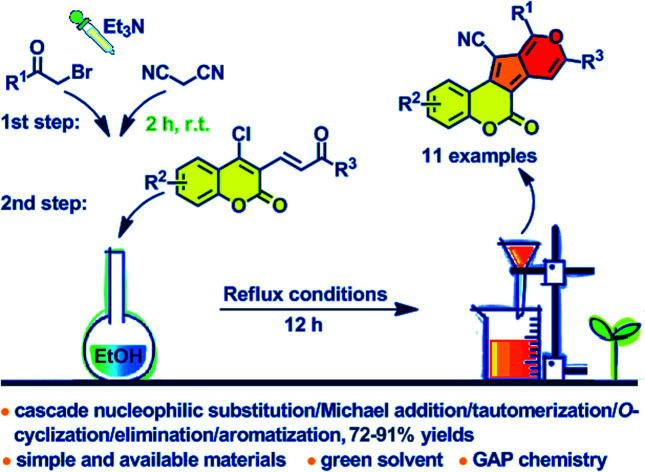

## Introduction

**Fig. 1:**
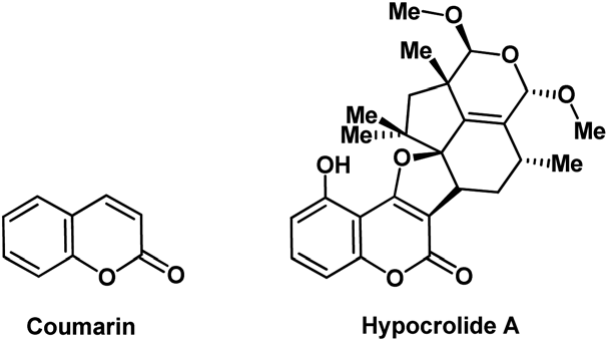
Structures of coumarin and hypocrolide A.

It is highly of interest to efficiently prepare various heterocyclic structures based on privileged frameworks from the point of view of synthetic organic chemistry and drug discovery. Coumarins are important privileged heterocycles because their derivatives are valuable structures for the discovery of novel pharmaceutically active molecules and therapeutic agents.^1–9^ For instance, hypocrolide A is a natural antibiotic,^10^ derived from the fungus *Hypocrea* sp, and contains the coumarin core (Fig. 1). Furthermore, some substituted coumarins are very favorable to use in perfumes, cosmetics, lasers, radiometric chemosensors, bio-sensors, and living cells imaging.^11–14^ Accordingly, the synthesis of molecules containing the coumarin motif is highly desirable.

**Fig. 2:**
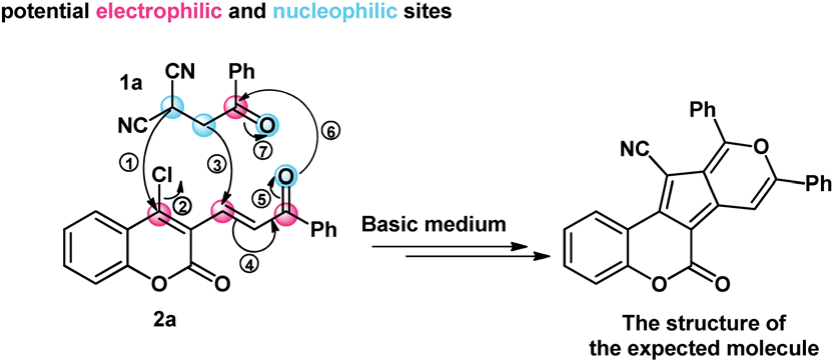
The potential active sites of the starting materials and the proposed strategy for the construction of coumarin-based cyclopenta[*c*]pyrans.

Based on the literature and our previous reports,^15–19^ 4-chloro-3-vinyl coumarin has three potential electrophilic active sites, which can selectively be attacked by various nucleophiles. Considering these active sites, we decided to investigate the base-mediated reaction of 4-chloro-3-vinyl coumarin 2a and β-ketodinitrile^20^1a as a bisnucleophilic synthon. Indeed, we envisioned that this designed reaction offers an efficient pathway for the structural unity between coumarin and cyclopenta[*c*]pyran^21–23^ moieties (Fig. 2).

**Fig. 3:**
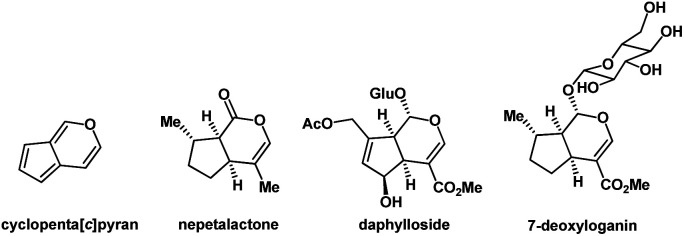
The core structure of cyclopenta[*c*]pyran and three natural iridoids possessing this substructure.

It is noteworthy that the cyclopenta[*c*]pyran scaffold is a privileged heterocyclic system that serves as the structural core in various functionalized natural products. For example, iridoids^24–28^ are an expansive family of natural monoterpenoids, which are characterized by their cyclopenta[*c*]pyran ring systems. The structures of some natural iridoids are shown in Fig. 3.

Members of this family have marine and terrestrial origins^29–31^ and they have attracted broad attention owing to their various pharmaceutical activities. In this context, developing straightforward methods for the preparation of such structures is worth exploring.

## Results and discussion

**Scheme 1:**
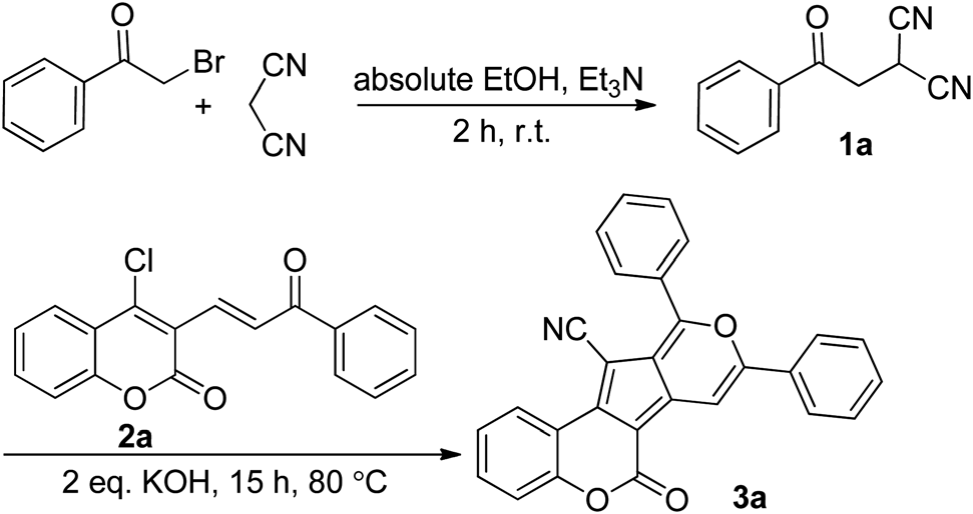
Synthesis of coumarin-based cyclopenta[*c*]pyran 3a.

We started our investigation with the preparation of β-ketodinitrile 1a by the base-mediated condensation of phenacyl bromide, and malononitrile in absolute EtOH at room temperature. After 2 h the reaction was completed, and compound 1a was formed. Then, without further purification within a one-pot sequential process, 4-chloro-3-vinyl coumarin 2a, and two equiv. KOH were added to the reaction tube. The reaction was stirred magnetically at room temperature for 24 h, but no specific product was formed. Pleasingly, a moderate yield of the expected coumarin-based cyclopenta[*c*]pyran 3a (55% yield) was obtained when the reaction mixture was refluxed at 80 °C for 15 hours (Scheme 1).

Encouraged by this very interesting result, the above reaction was chosen as the model reaction, and various bases were screened to optimize the reaction conditions. The desired product 3a was formed with moderate yields (49–60%) when inorganic bases such as NaOH, K_2_CO_3_, and Cs_2_CO_3_ were used (Table 1, entries 8–10). Then, piperidine (with relatively more nucleophilicity), DBU, and Et_3_N were tested for this process. The reaction promoted *via* piperidine didn't give 3a at all, probably owing to the nucleophilic substitution of piperidine with the chlorine atom of the substrate 2a (Table 1, entry 5). Moreover, product 3a was found with a negligible yield when DBU was used as base, and a mixture of overlapping spots were observed (Table 1, entry 6). Our examinations showed that Et_3_N is the best base for this transformation, affording the product 3a in a yield of 79% (Table 1, entry 1). In continue, different solvents were tested to obtain the optimal reaction solvent, and the best result was gained in absolute EtOH (Table 1, entries 1–4). It should be mentioned that the yield was remarkably decreased in non-anhydrous solvents. Furthermore, at temperatures below the reflux temperature, the reaction was completed over longer periods of time. We established the optimal reaction conditions for the preparation of coumarin-based cyclopenta[*c*]pyran derivatives as follows: use of 2.0 equiv. Et_3_N as the base and absolute EtOH as the solvent to perform the reaction at 80 °C (Table 1).

**Table 1:** Survey on conditions for the synthesis of 3a^a^

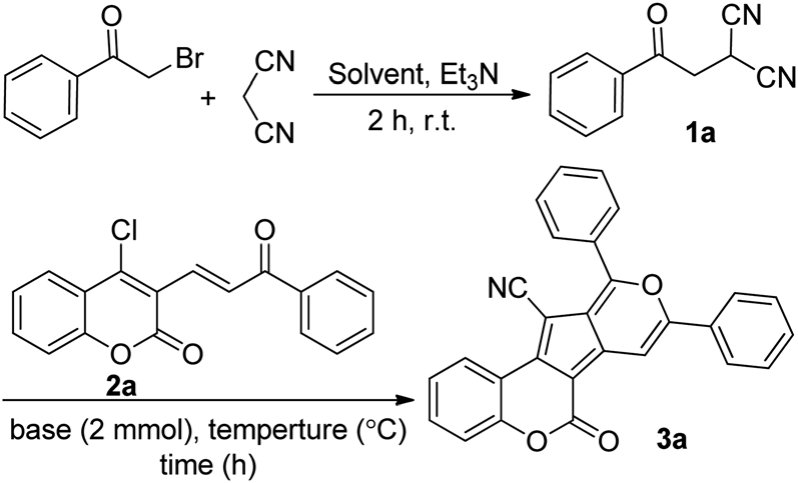					
Entry	Base	Solvent	Temp. (°C)	Time (h)	Yield (%)
1	Et_3_N	EtOH	80	12	79
2	Et_3_N	DMF	80	13	43
3	Et_3_N	MeCN	80	17	65
4	Et_3_N	THF	65	24	20
5	Piperidne	EtOH	80	—	—
6	DBU	EtOH	80	11	10
7	KOH	EtOH	80	15	55
8	NaOH	EtOH	80	16	49
9	Cs_2_CO_3_	EtOH	80	10	60
10	K_2_CO_3_	EtOH	80	13	57

a To a magnetically stirred solution of phenacyl bromide (1 mmol, 199 mg), and malononitrile (1 mmol, 66 mg), was added Et_3_N (1 mmol, 101 mg) in the mentioned absolute solvent. After 2 h, substrate 2a (1 mmol, 310 mg), and base (2 mmol) were added to the reaction mixture. The reaction was carried out at mentioned temperature, and it was monitored by TLC. After above mentioned time a brilliant orange product was isolated by filtration, and purified by washing with EtOH (96%).

**Scheme 2:**
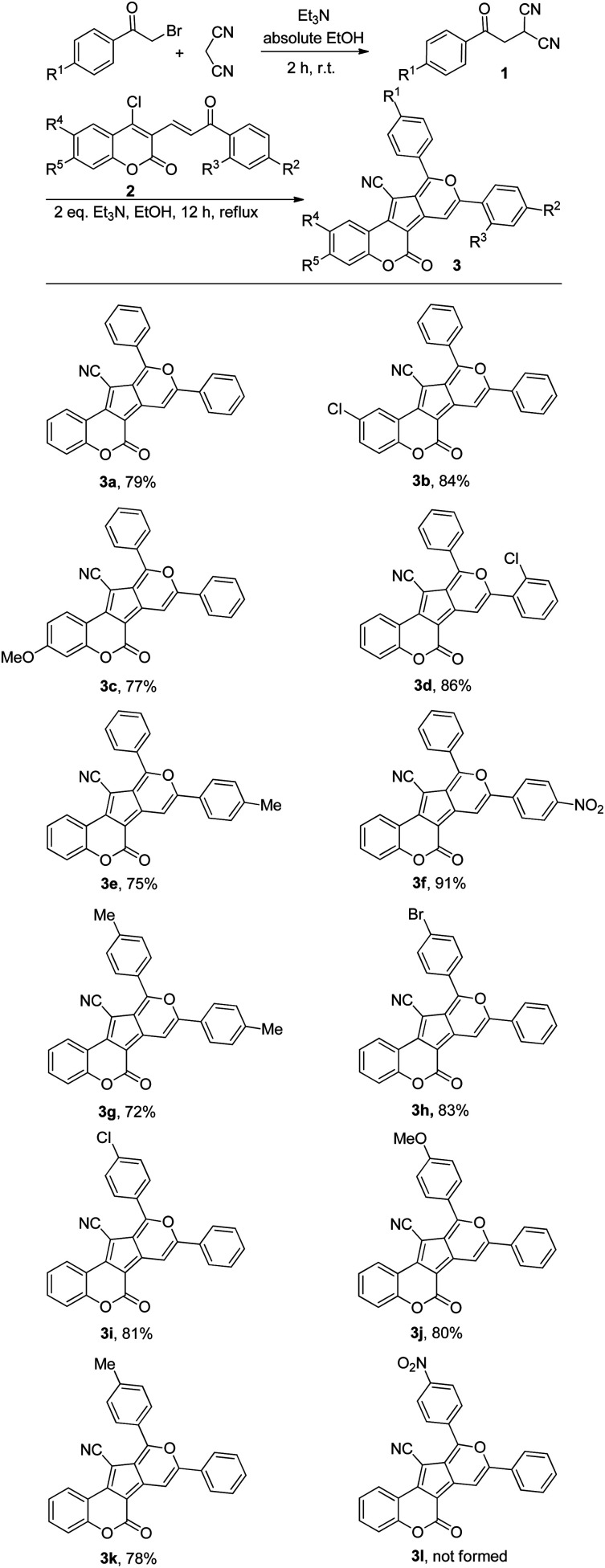
Synthesis of coumarin-based cyclopenta[*c*]pyrans.

With the optimized reaction conditions for the synthesis of coumarin-based cyclopenta[*c*]pyrans in hand, we set out to investigate the generality of this cascade transformation using differently substituted α,β-unsaturated coumarins. Electron-donating and – withdrawing substitutes on aromatic rings of substrate 2 were all tolerated, affording the expected coumarin-based cyclopenta[*c*]pyrans (Scheme 2) in satisfactory yields (75–91%, 3b–3f). It is worth mentioning here that the nature of the substituent on α,β-unsaturated coumarins had a slight impact on the yields. Next, the influence of different substituents (either electron-withdrawing or electron-donating) of β-ketodinitrile onto this process was investigated, and products 3g–3k were obtained in very good yields (72–83%). Notably, for substrate 1 with a NO_2_ substituent attached on the benzene ring, no expected product was detected. With the aim of exploring the synthetic utility of this novel domino process, a gram-scale experiment was performed with phenacyl bromide (3.5 mmol, 0.696 g), malononitrile (3.5 mmol, 0.233 g), and 4-chloro-3-3-viyl coumarin (3.5 mmol, 1.08 g), yielding the desired compound 3a in 71% yield without a remarkable loss of efficiency compared to small scale (79%).

**Fig. 4:**
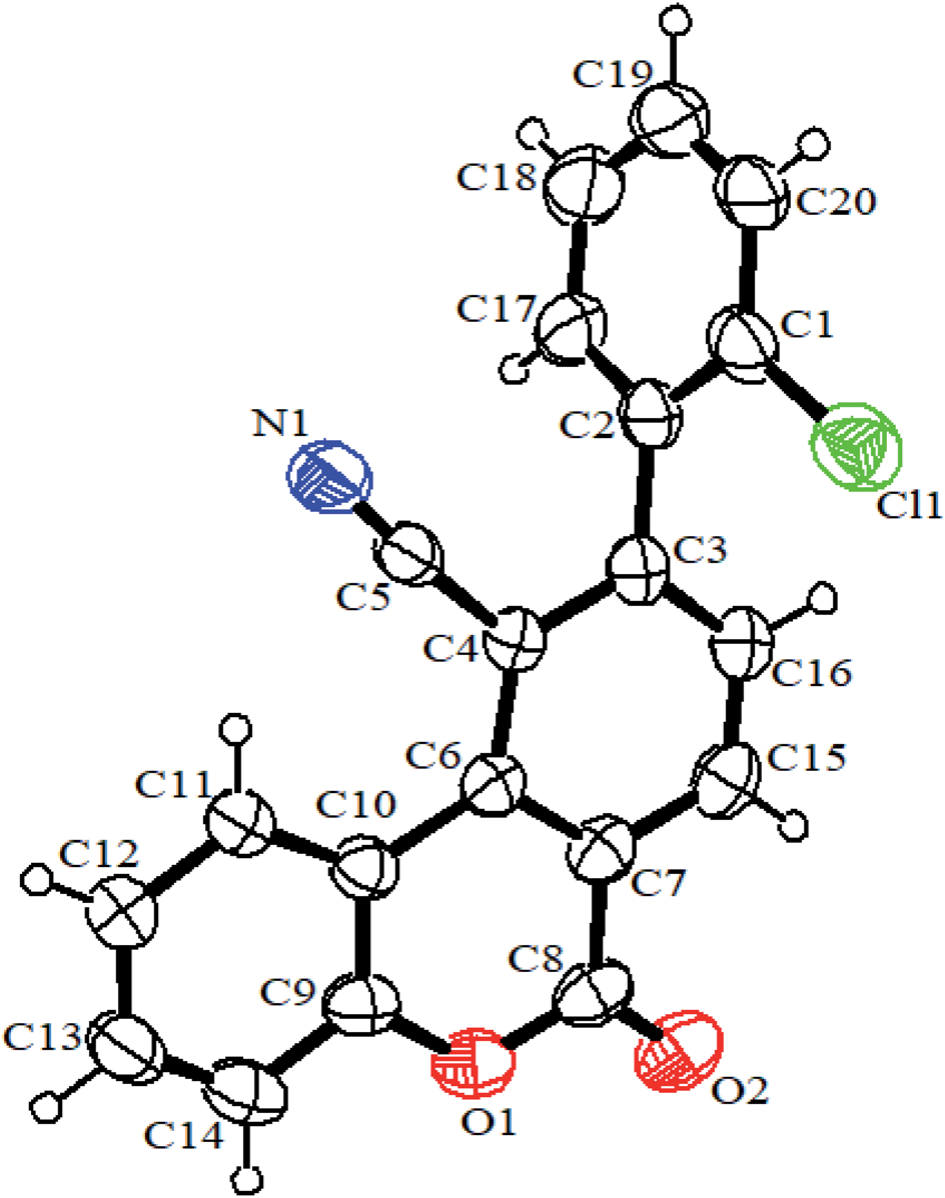
ORTEP diagram of 3d (CCDC 1955485).

The structures of the synthesized products were characterized by Fourier transform infrared (FTIR), mass spectrometry, elemental analysis, and ^1^H NMR. Notably, the solubility of the products was too low, and except for 3d which was more soluble than the others, we couldn't record ^13^C{H} NMR spectra for them. The molecular structures of the synthesized compounds were undeniably confirmed by X-ray crystallographic analysis of product 3d (Fig. 4).

**Scheme 3:**
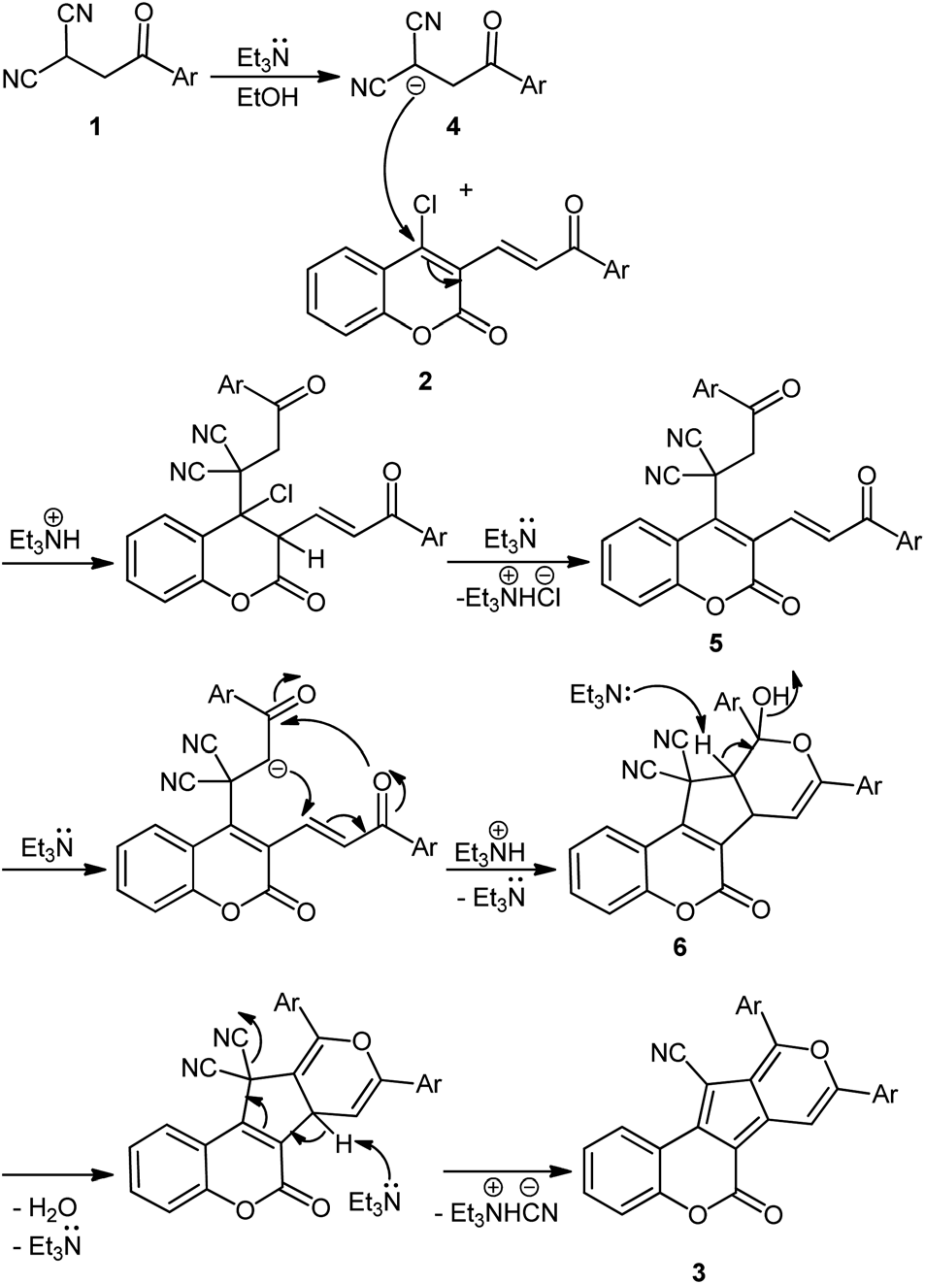
Mechanistic rationalization for the preparation of 3.

The mechanism of the first step including β-ketodinitriles formation is known.^32–38^ Based on experimental observations, a plausible mechanism for the formation of coumarin-based cyclopenta[*c*]pyrans is depicted in Scheme 3. Iinitially, Et_3_N deprotonates β-ketodinitrile 1 to form anion intermediate 4. Nucleophilic addition of the intermediate 4 to substrate 2, followed by base-mediated liberation of HCl afford intermediate 5. After that, a domino base-assisted intramolecular Michael addition and subsequent *O*-cyclization occurs and intermediate 6 forms. The final aromatic product 3 is generated by the base-mediated elimination of H_2_O and HCN.

## Conclusions

In summary, we presented a novel domino-type process for the synthesis of substituted coumarin-based cyclopenta[*c*]pyrans through a base-mediated nucleophilic substitution/Michael addition/tautomerization/*O*-cyclization/elimination/aromatization reaction of 4-chloro-3-vinyl coumarins with β-ketodinitriles. Notably, two new carbon–carbon bonds and one carbon–oxygen bond were formed in these reactions, and highly stable polycyclic aromatic products were obtained. This unprecedented strategy was carried out under relatively green conditions, providing an efficient approach to structural unity between two valuable organic moieties.

## Experimental

### General

Two melting points were measured on an Electrothermal 9100 apparatus. IR spectra were recorded as KBr pellets on a Nicolet FTIR 100 spectrophotometer. ^1^H NMR (500 MHz, 300 MHz) and ^13^C NMR (75 MHz) spectra were obtained using Bruker DRX-500 Avance and Bruker DRX-300 Avance spectrometers. All NMR spectra were recorded at r.t. in DMSO-*d*_6_ and CDCl_3_. Chemical shifts are reported in parts per million (*δ*) downfield from an internal TMS reference. Coupling constants (*J* values) are reported in hertz (Hz), and standard abbreviations were used to indicate spin multiplicities. Elemental analyses for C, H, and N were performed using a Heraeus CHN-O-Rapid analyzer. Mass spectra were recorded on a Finnigan-MATT 8430 mass spectrometer operating at an ionization potential of 70 eV. All chemicals and solvents were purchased from Merck or Aldrich and were used without further purification. Starting materials were synthesized according to the procedures reported in the literature.^15–20^ Single crystals of compounds 3d were formed in CH_2_Cl_2_.

### General procedure for the preparation of compounds 3a–3k

To a magnetically stirred solution of phenacyl bromide (1 mmol, 199 mg), and malononitrile (1 mmol, 66 mg), was added Et_3_N (1 mmol, 101 mg) in the absolute EtOH (5 ml). After 2 h, α,β-unsaturated coumarin 2 (1 mmol, 310 mg), and Et_3_N (2 mmol) were added to the reaction mixture. The reaction was carried out at 80 °C, and it was monitored by TLC. After 12 h a brilliant orange product was isolated by filtration, and purified by washing with EtOH (96%).

#### 6-Oxo-8,10-diphenyl-6*H*-pyrano[3′,4′:4,5]cyclopenta[1,2-*c*]chromene-11-carbonitrile (3a)

Orange powder, dec. point = 310–312 °C, 0.32 g, yield: 79%. IR (KBr): 2201 (C≡N), 1720 (C=O), 1643, 1601, and 1542 (Ar), 1225, 1177, 1114, and 999 (C–O) cm^−1^. Anal. calcd for C_28_H_15_NO_3_ (413.42): C, 81.35; H, 3.66, N, 3.39%. Found: C, 81.34; H, 3.62, N, 3.36%. ^1^H NMR (500 MHz, DMSO-*d*_6_): *δ* = 7.46 (1H, t, ^3^*J*_HH_ = 8.5 Hz, CH_2_ of coumarin), 7.47 (1H, d, ^3^*J*_HH_ = 8.3 Hz, CH_4_ of coumarin), 7.63–7.66 (4H, m, 4CH of Ph), 7.77 (2H, t, ^3^*J*_HH_ = 7.2 Hz, 2CH_*para*_ of Ph), 7.83 (1H, t, ^3^*J*_HH_ = 7.7 Hz, CH_2_ of coumarin), 8.15 (2H, t, ^3^*J*_HH_ = 7.2 Hz, 2CH_*ortho*_ of Ph), 8.17 (2H, t, ^3^*J*_HH_ = 7.2 Hz, 2CH_*ortho*_ of Ph), 8.55 (1H, d, ^3^*J*_HH_ = 7.8 Hz, CH_1_ of coumarin), 8.62 (1H, s, CH_7_). MS (ESI, 70 eV): *m*/*z* (%) = 414 (M^+^, 100), 384 (12), 356 (24), 327 (24), 251 (22), 206 (18), 105 (13), 77 (16).

#### 2-Chloro-6-oxo-8,10-diphenyl-6*H*-pyrano[3′,4′:4,5]cyclopenta[1,2-*c*]chromene-11-carbonitrile (3b)

Orange powder, dec. point = 318–320 °C, 0.37 g, yield: 84%. IR (KBr): 2202 (C≡N), 1730 (C=O), 1615, 1555, 1541, and 1468 (Ar), 1228, 1176, and 1005 (C–O) cm^−1^. Anal. calcd for C_28_H_14_ClNO_3_ (447.87): C, 75.09; H, 3.15, N, 3.13%. Found C, 75.07; H, 3.12, N, 3.12%. ^1^H NMR (300 MHz, CDCl_3_), *δ* = 7.35 (1H, d, ^3^*J*_HH_ = 8.8 Hz, CH_2_ of coumarin), 7.49 (1H, dd, ^3^*J*_HH_ = 8.8 Hz, ^2^*J*_HH_ = 2.5 Hz, CH_4_ of coumarin), 7.56–7.60 (3H, m, 3CH of Ph), 7.73–7.76 (3H, m, 3CH of Ph), 8.07 (2H, t, ^3^*J*_HH_ = 6.2 Hz, 2CH_*ortho*_ of Ph), 8.09 (2H, t, ^3^*J*_HH_ = 6.2 Hz, 2CH_*ortho*_ of Ph), 8.66 (1H, d, ^3^*J*_HH_ = 2.6 Hz, CH_1_ of coumarin), 8.74 (1H, s, CH_7_). MS (ESI, 70 eV): *m*/*z* (%) = 447 (M^+^, 100), 427 (3), 390 (6), 327 (13), 251 (9), 105 (11), 77 (14).

#### 3-Methoxy-6-oxo-8,10-diphenyl-6*H*-pyrano[3′,4′:4,5]cyclopenta[1,2-*c*]chromene-11-carbonitrile (3c)

Orange powder, dec. point = 343–345 °C, 0.34 g, yield: 77%. IR (KBr): 2198 (C≡N), 1727 (C=O), 1614, 1556, 1460, and 1421 (Ar), 1205, 1166, 1117, and 1035 (C–O) cm^−1^. Anal. calcd for C_29_H_17_NO_4_ (443.45): C, 78.55; H, 3.86, N, 3.16%. Found: C, 78.54; H, 3.85, N, 3.14%. ^1^H NMR (500 MHz, CDCl_3_): *δ* = 3.92 (3H, s, OCH_3_), 6.92 (1H, d, ^3^*J*_HH_ = 2.5 Hz, CH_4_ of coumarin), 6.95 (1H, dd, ^3^*J*_HH_ = 8.5 Hz, ^2^*J*_HH_ = 2.5 Hz, CH_2_ of coumarin), 7.55–7.60 (3H, m, 3CH of Ph), 7.72–7.75 (3H, m, 3CH of Ph), 8.07 (4H, d, ^3^*J*_HH_ = 8.7 Hz 4CH_*ortho*_ of Ph), 8.66 (1H, d, ^3^*J*_HH_ = 8.5 Hz, CH_1_ of coumarin), 8.74 (1H, s, CH_7_). MS (ESI, 70 eV): *m*/*z* (%) = 443 (M^+^, 100), 400 (20), 372 (10), 314 (18), 221 (23), 105 (38), 77 (24).

#### 8-(2-Chlorophenyl)-6-oxo-10-phenyl-6*H*-pyrano[3′,4′:4,5]cyclopenta[1,2-*c*]chromene-11-carbonitrile (3d)

Orange powder, dec. point = 255–257 °C, 0.38 g, yield: 86%. IR (KBr): 2204 (C≡N), 1706 (C=O), 1605, 1548, and 1468 (Ar), 1176, 1115, 1045, and 1029 (C–O) cm^−1^. Anal. calcd for C_28_H_14_ClNO_3_ (447.87): C, 75.09; H, 3.15, N, 3.13%. Found: C, 75.06; H, 3.12, N, 3.12%. ^1^H NMR (500 MHz, DMSO-*d*_6_): *δ* = 7.42 (1H, t, ^3^*J*_HH_ = 7.4 Hz, CH_2_ of coumarin), 7.47 (1H, d, ^3^*J*_HH_ = 8.0 Hz, CH_4_ of coumarin), 7.59–7.66 (3H, m, 3CH of Ar), 7.70–7.74 (3H, m, 3CH of Ph), 7.78 (1H, t, ^3^*J*_HH_ = 7.4 Hz, CH_3_ of coumarin), 7.98 (1H, d, ^3^*J*_HH_ = 7.0 Hz, CH of Ar), 8.11 (2H, d, ^3^*J*_HH_ = 7.2 Hz, 2CH of Ar), 8.50 (1H, d, ^3^*J*_HH_ = 7.8 Hz, CH_1_ of coumarin), 8.51 (1H, s, CH_7_). ^13^C NMR (75 MHz, CDCl_3_): *δ* = 80.76, 111.48, 117.00, 117.70, 117.94, 123.74, 124.72, 125.59, 127.49, 128.86, 129.77, 130.77, 131.01, 131.09, 131.10, 131.47, 131.97, 132.20, 133.07, 139.31, 148.03, 153.31, 155.44, 158.49, 162.77. MS (ESI, 70 eV): *m*/*z* (%) = 447 (M^+^, 7), 308 (13), 251 (11), 139 (100), 105 (46), 77 (34). Crystal data for 3d C_28_H_14_ClNO_3_ (CCDC 1955485): *M*_W_ = 575.55, monoclinic, *P*121/*n*1, *a* = 7.4936(15) Å, *b* = 24.222(5) Å, *c* = 13.519(3) Å, *α* = 90, *β* = 101.90(3), *γ* = 90, *V* = 2401.1(9) Å^3^, *Z* = 4, *D*_c_ = 1.474 mg m^−3^, *F*(000) = 1088, crystal dimension 0.50 × 0.30 × 0.20 mm, radiation, Mo Kα (*λ* = 0.71073 Å), 2.280 ≤ 2*θ* ≤ 24.499, intensity data were collected at 293(2) K with a Bruker APEX area-detector diffractometer, and employing *ω*/2*θ* scanning technique, in the range of −8 ≤ *h* ≤ 8, 0 ≤ *k* ≤ 28, 0 ≤ *l* ≤ 15; the structure was solved by a direct method, all non-hydrogen atoms were positioned and anisotropic thermal parameters refined from 3827 observed reflections with *R* (into) = 0.0286 by a full-matrix least-squares technique converged to *R*1 = 0.0695, and w*R*_2_ = 0.1724 [*I* > 2sigma(*I*)].

#### 6-Oxo-8-phenyl-10-(*p*-tolyl)-6*H*-pyrano[3′,4′:4,5]cyclopenta[1,2-*c*]chromene-11-carbonitrile (3e)

Orange powder, dec. point = 342–345 °C, 0.32 g, yield: 75%. IR (KBr): 2198 (C≡N), 1730 (C=O), 1605, 1560, 1543, and 1511 (Ar), 1176, 1112, 1052, and 1036 (C–O) cm^−1^. Anal. calcd for C_29_H_17_NO_3_ (427.45): C, 81.49; H, 4.01, N, 3.28%. Found: C, 81.44; H, 4.02, N, 3.26%. ^1^H NMR (300 MHz, DMSO-*d*_6_): *δ* = 2.41 (3H, s, CH_3_), 7.44 (2H, d, ^3^*J*_HH_ = 8.0 Hz, 2CH of Ar), 7.45 (1H, t, ^3^*J*_HH_ = 7.8 Hz, CH_2_ of coumarin), 7.49 (1H, d, ^3^*J*_HH_ = 7.8 Hz, CH_4_ of coumarin), 7.67 (1H, t, ^3^*J*_HH_ = 7.4 Hz, CH_3_ of coumarin), 7.74 (1H, t, ^3^*J*_HH_ = 8.5 Hz, CH_*para*_ of Ph), 7.77 (2H, t, ^3^*J*_HH_ = 8.5 Hz, 2CH_*meta*_ of Ph), 8.05 (2H, d, ^3^*J*_HH_ = 8.0 Hz, 2CH of Ar), 8.15 (2H, d, ^3^*J*_HH_ = 8.4 Hz, 2CH_*ortho*_ of Ph), 8.58 (1H, d, ^3^*J*_HH_ = 7.7 Hz, CH_1_ of coumarin), 8.62 (1H, s, CH_7_). MS (ESI, 70 eV): *m*/*z* (%) = 427 (M^+^, 100), 370 (6), 354 (8), 340 (10), 327 (14), 264 (12), 105 (55), 77 (21).

#### 8-(4-Nitrophenyl)-6-oxo-10-phenyl-6*H*-pyrano[3′,4′:4,5]cyclopenta[1,2-*c*]chromene-11-carbonitrile (3f)

Orange powder, dec. point = 367–369 °C, 0.41 g, yield: 91%. IR (KBr): 2204 (C≡N), 1720 (C=O), 1594, 1542, 1521, and 1424 (Ar), 1175, 1111, 1051, and 1000 (C–O) cm^−1^. Anal. calcd for C_28_H_14_N_2_O_5_ (458.09): C, 73.36; H, 3.08, N, 6.11%. Found: C, 73.34; H, 3.10, N, 6.13%. ^1^H NMR (500 MHz, DMSO-*d*_6_): *δ* = 7.43 (1H, t, ^3^*J*_HH_ = 8.4 Hz, CH_2_ of coumarin), 7.46 (1H, d, ^3^*J*_HH_ = 8.6 Hz, CH_4_ of coumarin), 7.63 (1H, t, ^3^*J*_HH_ = 8.0 Hz, CH_3_ of coumarin), 7.76 (2H, t, ^3^*J*_HH_ = 7.6 Hz, 2CH_*meta*_ of Ph), 7.82 (1H, t, ^3^*J*_HH_ = 7.4 Hz, CH_*para*_ of Ph), 8.17 (2H, d, ^3^*J*_HH_ = 7.6 Hz, 2CH_*ortho*_ of Ph), 8.38 (2H, d, ^3^*J*_HH_ = 8.6 Hz, 2CH of Ar), 8.41 (2H, d, ^3^*J*_HH_ = 8.6 Hz, 2CH of Ar), 8.55 (1H, d, ^3^*J*_HH_ = 7.9 Hz, CH_1_ of coumarin), 8.80 (1H, s, CH_7_). MS (ESI, 70 eV): *m*/*z* (%) = 458 (M^+^, 100), 412 (23), 354 (30), 327 (62), 251 (28), 105 (71), 77 (46).

#### 6-Oxo-8,10-di-*p*-tolyl-6*H*-pyrano[3′,4′:4,5]cyclopenta[1,2-*c*]chromene-11-carbonitrile (3g)

Orange powder, dec. point = 348–350 °C, 0.31 g, yield: 72%. IR (KBr): 2198 (C≡N), 1730 (C=O), 1604, 1563, 1484, and 1422 (Ar), 1175, 1112, 1051, and 998 (C–O) cm^−1^. Anal. calcd for C_30_H_19_NO_3_ (441.14): C, 81.62; H, 4.34, N, 3.17%. Found: C, 81.63; H, 4.36, N, 3.15%. ^1^H NMR (500 MHz, DMSO-*d*_6_): *δ* = 2.47 (3H, s, CH_3_), 2.55 (3H, s, CH_3_), 7.37 (2H, d, ^3^*J*_HH_ = 7.7 Hz, 2CH of Ar), 7.38 (1H, t, ^3^*J*_HH_ = 7.2 Hz, CH_2_ of coumarin), 7.43 (1H, d, ^3^*J*_HH_ = 8.2 Hz, CH_4_ of coumarin), 7.53 (2H, d, ^3^*J*_HH_ = 7.7 Hz, 2CH of Ar), 7.56 (1H, t, ^3^*J*_HH_ = 7.7 Hz, CH_3_ of coumarin), 7.96 (2H, d, ^3^*J*_HH_ = 7.8 Hz, 2CH of Ar), 7.99 (2H, d, ^3^*J*_HH_ = 7.9 Hz, 2CH of Ar), 8.72 (1H, s, CH_7_), 8.77 (1H, d, ^3^*J*_HH_ = 7.9 Hz, CH_1_ of coumarin). MS (ESI, 70 eV): *m*/*z* (%) = 441 (M^+^, 100), 354 (16), 264 (19), 220 (29), 119 (25), 91 (57).

#### 10-(4-Bromophenyl)-6-oxo-8-phenyl-6*H*-pyrano[3′,4′:4,5]cyclopenta[1,2-*c*]chromene-11-carbonitrile (3h)

Orange powder, dec. point = 357–359 °C, 0.40 g, yield: 83%. IR (KBr): 2204 (C≡N), 1729 (C=O), 1602, 1563, 1541, and 1425 (Ar), 1175, 1113, 1051, and 1005 (C–O) cm^−1^. Anal. calcd for C_28_H_14_BrNO_3_ (491.02): C, 68.31; H, 2.87, N, 2.85%. Found: C, 68.33; H, 2.85, N, 2.86%. ^1^H NMR (500 MHz, DMSO-*d*_6_): *δ* = 7.48 (1H, t, ^3^*J*_HH_ = 8.2 Hz, CH_2_ of coumarin), 7.49 (1H, d, ^3^*J*_HH_ = 8.4 Hz, CH_4_ of coumarin), 7.63 (2H, d, ^3^*J*_HH_ = 7.0 Hz, 2CH of Ar), 7.64 (1H, t, ^3^*J*_HH_ = 8.2 Hz, CH_*para*_ of Ph), 7.67 (1H, t, 7.2 Hz, CH_3_ of coumarin), 7.98 (2H, d, ^3^*J*_HH_ = 8.2 Hz, 2CH_*meta*_ of Ph), 8.11 (2H, d, ^3^*J*_HH_ = 8.2 Hz, 2CH_*ortho*_ of Ph), 8.16 (2H, d, ^3^*J*_HH_ = 7.0 Hz, 2CH of Ar), 8.56 (1H, d, ^3^*J*_HH_ = 7.0 Hz, CH_1_ of coumarin), 8.66 (1H, s, CH_7_). MS (ESI, 70 eV): *m*/*z* (%) = 493 (M^+^ + 1, 100), 491 (M^+^, 98), 327 (26), 251 (30), 105 (27), 77 (41).

#### 10-(4-Chlorophenyl)-6-oxo-8-phenyl-6*H*-pyrano[3′,4′:4,5]cyclopenta[1,2-*c*]chromene-11-carbonitrile (3i)

Orange powder, dec. point = 370–373 °C, 0.36 g, yield: 81%. IR (KBr): 2200 (C≡N), 1729 (C=O), 1602, 1563, 1468, and 1423 (Ar), 1225, 1175, 1112, and 1051 (C–O) cm^−1^. Anal. calcd for C_28_H_14_ClNO_3_ (447.87): C, 75.09; H, 3.15, N, 3.13%. Found: C, 75.06; H, 3.13, N, 3.12%. ^1^H NMR (500 MHz, CDCl_3_): *δ* = 7.42 (1H, t, ^3^*J*_HH_ = 7.3 Hz, CH_2_ of coumarin), 7.46 (1H, d, ^3^*J*_HH_ = 8.2 Hz, CH_4_ of coumarin), 7.50–7.64 (4H, m, 2CH_*meta*_ of Ph, CH_*para*_ of Ph, and CH_3_ of coumarin), 7.74 (2H, d, ^3^*J*_HH_ = 8.5 Hz, 2CH of Ar), 8.06 (2H, d, ^3^*J*_HH_ = 8.5 Hz, 2CH of Ar), 8.09 (2H, d, ^3^*J*_HH_ = 8.0 Hz, 2CH_*meta*_ of Ph), 8.78 (1H, d, ^3^*J*_HH_ = 7.9 Hz, CH_1_ of coumarin), 8.81 (1H, s, CH_7_). MS (ESI, 70 eV): *m*/*z* (%) = 447 (M^+^, 2), 327 (10), 308 (9), 251 (18), 138 (100), 105 (31), 77 (28).

#### 8-(4-Methoxyphenyl)-6-oxo-10-phenyl-6*H*-pyrano[3′,4′:4,5]cyclopenta[1,2-*c*]chromene-11-carbonitrile (3j)

Orange powder, dec. point = 318–320 °C, 0.35 g, yield: 80%. IR (KBr): 2198 (C≡N), 1735 (C=O), 1603, 1559, 1547, and 1470 (Ar), 1000 (C–O) cm^−1^. Anal. calcd for C_29_H_17_NO_4_ (443.45): C, 78.55; H, 3.86, N, 3.16%. Found: C, 78.54; H, 3.84, N, 3.15%. ^1^H NMR (500 MHz, DMSO-*d*_6_): *δ* = 3.95 (3H, s, OCH_3_), 7.28 (2H, d, ^3^*J*_HH_ = 8.7 Hz, 2CH of Ar), 7.40 (1H, t, ^3^*J*_HH_ = 7.1 Hz, CH_4_ of coumarin), 7.41 (1H, d, ^3^*J*_HH_ = 7.1 Hz, CH_2_ of coumarin), 7.50–7.60 (3H, m, 3CH of Ph), 7.60 (1H, t, ^3^*J*_HH_ = 6.8 Hz, CH_3_ of coumarin), 8.07 (2H, d, ^3^*J*_HH_ = 7.3 Hz, 2CH_*ortho*_ of Ph), 8.11 (2H, d, ^3^*J*_HH_ = 8.6 Hz, 2CH of Ar), 8.48 (1H, s, CH_7_), 8.51 (1H, d, ^3^*J*_HH_ = 7.0 Hz, CH_1_ of coumarin). MS (ESI), 70 eV: *m*/*z* (%) = 443 (M^+^, 100), 428 (2), 372 (7), 344 (7), 314 (9), 221 (16), 105 (20), 77 (13).

#### 6-Oxo-8-phenyl-10-(*p*-tolyl)-6*H*-pyrano[3′,4′:4,5]cyclopenta[1,2-*c*]chromene-11-carbonitrile (3k)

Orange powder, dec. point = 301–305 °C, 0.33 g, yield: 78%. IR (KBr): 2181 (C≡N), 1680 (C=O), 1652, 1616, 1598, and 1486 (Ar), 1218, 1201, 1169, and 1105 (C–O) cm^−1^. Anal. calcd for C_29_H_17_NO_3_ (427.12): C, 81.49; H, 4.01, N, 3.28%. Found: C, 81.51; H, 4.02, N, 3.28%. ^1^H NMR (500 MHz, DMSO-*d*_6_): *δ* = 2.56 (3H, s, CH_3_), 7.38 (1H, t, ^3^*J*_HH_ = 7.6 Hz, CH_2_ of coumarin), 7.43 (1H, d, ^3^*J*_HH_ = 8.3 Hz, CH_2_ of coumarin), 7.52–7.60 (6H, m, 6CH of Ar), 8.00 (2H, d, ^3^*J*_HH_ = 7.7 Hz, 2CH_*ortho*_ of Ph), 8.07 (2H, d, ^3^*J*_HH_ = 7.5 Hz, 2CH of Ar), 8.77 (1H, s, CH_7_), 8.78 (1H, d, ^3^*J*_HH_ = 7.9 Hz, CH_1_ of coumarin). MS (ESI), 70 eV: *m*/*z* (%) = 427 (M^+^, 100), 370 (4), 340 (5), 327 (4), 213 (9).

## Conflicts of interest

There are no conflicts to declare.

## Supplementary Material

RA-012-D2RA00594H-s001

RA-012-D2RA00594H-s002
