# Correction: Chemoselective and one-pot synthesis of novel coumarin-based cyclopenta[*c*]pyrans *via* base-mediated reaction of α,β-unsaturated coumarins and β-ketodinitriles

**DOI:** 10.1039/d2ra90023h

**Published:** 2022-03-17

**Authors:** Behnaz Farajpour, Abdolali Alizadeh

**Affiliations:** Department of Chemistry, Tarbiat Modares University P. O. Box 14115-175 Tehran Iran

## Abstract

Correction for ‘Chemoselective and one-pot synthesis of novel coumarin-based cyclopenta[*c*]pyrans *via* base-mediated reaction of α,β-unsaturated coumarins and β-ketodinitriles’ by Behnaz Farajpour *et al.*, *RSC Adv.*, 2022, **12**, 7262–7267, DOI: 10.1039/D2RA00594H.

The authors regret that an incorrect version of Fig. 4 was included in the original article. The correct version of Fig. 4 is presented below. The CCDC number was also incorrectly cited for this same compound (compound **3d**) and should instead be cited as 1970237.

**Fig. 1:**
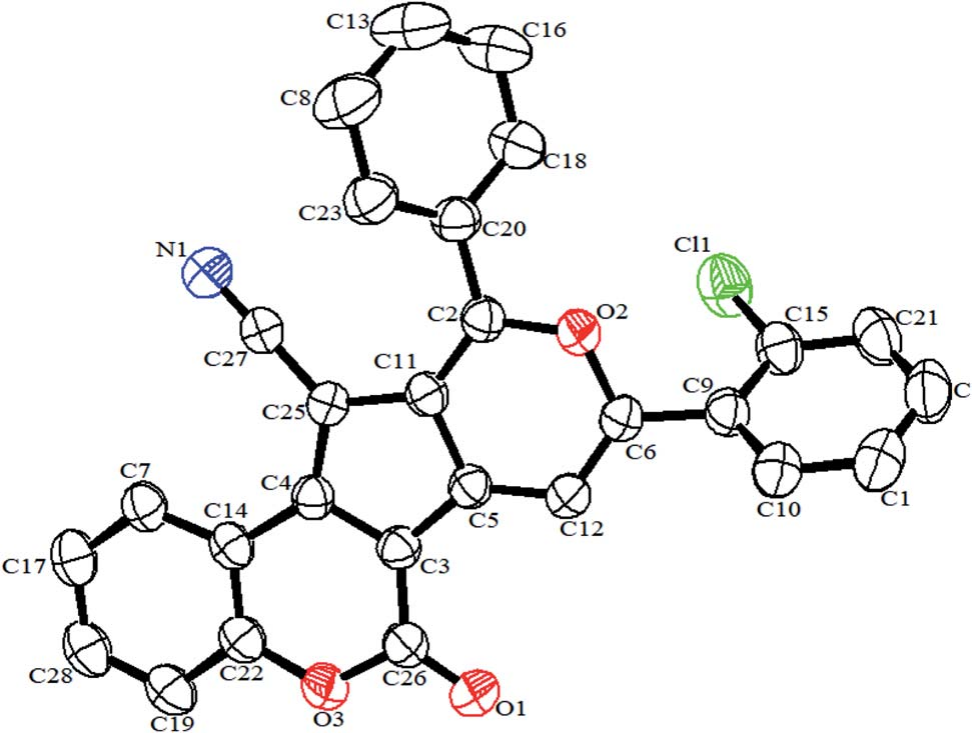
ORTEP diagram of **3d** (CCDC 1970237).

The Royal Society of Chemistry apologises for these errors and any consequent inconvenience to authors and readers.

## Supplementary Material

