# Supplementary material for: Chemoselective and one-pot synthesis of novel coumarin-based cyclopenta[c]pyrans via base-mediated reaction of α,β-unsaturated coumarins and β-ketodinitriles
Source: RSC Adv. 2022 Mar 4;12(12):7262–7. doi: 10.1039/d2ra00594h (PMC8982290; doi:10.1039/d2ra00594h)
Supplement: RA-012-D2RA00594H-s001 [file RA-012-D2RA00594H-s001.pdf]

Electronic Supplementary Material (ESI) for RSC Advances.
This journal is © The Royal Society of Chemistry 2022

Chemoselective Synthesis of Novel Coumarin-based Cyclopenta[*c*]pyrans *via* Base-mediated Reaction of *α,β*-Unsaturated Coumarins and *β*-Ketodinitriles

Behnaz Farajpour, Abdolali Alizadeh*

Department of Chemistry, Tarbiat Modares University, P. O. Box 14115-175, Tehran, Iran

09/03/2022, ESI first published: 04/03/2022

This version of the Electronic Supplementary Information replaces a previous copy in which the crystallographic details for compound 3d was incorrect.

The Table of Contents

Title, author's name, address and Table of contents	S1
General procedure for preparation of **3a-3k**	S2
Characteristic data for compounds **3a-3k**	S2-S11
X-ray data and ortep diagram of compound **3d**	S6
IR, ^{1}H, ^{13}C NMR and Mass spectra of **3a-3k**	S12-S45

Experimental section

General

Keywords: 4-chloro-3-vinyl coumarins, 2-(2-oxo-2-phenylethyl)malononitriles *β*-ketodinitriles, *α,β*-unsaturated coumarins, coumarin-based cyclopenta[*c*]pyrans.

*Corresponding author, Tel.: +98 21 8800663; fax: +98 21 88006544; e-mail: aalizadeh@modares.ac.ir

Melting points were measured on an Electrothermal 9100 apparatus. IR spectra were recorded as KBr pellets on a Nicolet FTIR 100 spectrophotometer. ^{1}H NMR (500 MHz, 300 MHz) and ^{13}C NMR (75 MHz) spectra were obtained using Bruker DRX-500 Avance and Bruker DRX-300 Avance spectrometers. All NMR spectra were recorded at r.t. in DMSO-d_6 and $CDCl_3$. Chemical shifts are reported in parts per million (δ) downfield from an internal TMS reference. Coupling constants (J values) are reported in hertz (Hz), and standard abbreviations were used to indicate spin multiplicities. Elemental analyses for C, H, and N were performed using a Heraeus CHN-O-Rapid analyzer. Mass spectra were recorded on a Finnigan-MATT 8430 mass spectrometer operating at an ionization potential of 70 eV. All chemicals and solvents were purchased from Merck or Aldrich and were used without further purification. Starting materials were synthesized according to the procedures reported in the literature. Single crystals of compounds **3d** were formed in CH_2Cl_2.

General procedure for the preparation of 3a-3g.

To a magnetically stirred solution of phenacyl bromide (1 mmol, 199 mg), and malononitrile (1 mmol, 66 mg), was added Et_3N (1 mmol, 101 mg) in absolute EtOH (5 ml). After 2 h, α,β-unsaturated coumarin **2** (1 mmol, 310 mg), and Et_3N (2 mmol) were added to the reaction mixture. The reaction was carried out at 80 ºC, and it was monitored by TLC. After 12 h a brilliant orange product was isolated by filtration, and purified by washing with EtOH (96%).

Characteristic data for compounds 3a-3g.

6-Oxo-8,10-diphenyl-6*H*-pyrano[3',4':4,5]cyclopenta[1,2-*c*]chromene-11-carbonitrile (3a).

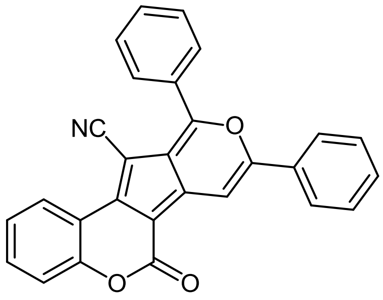

Orange powder, dec point = 310-312 °C, 0.32 g, yield: 79%. IR (KBr): 2201 (C≡N), 1720 (C=O), 1643, 1601, and 1542 (Ar), 1225, 1177, 1114, and 999 (C-O) cm^{-1}. Anal. calcd. for $C_{28}H_{15}NO_3$ (413.42): C, 81.35; H, 3.66, N, 3.39%. Found: C, 81.34; H, 3.62, N, 3.36%. ^{1}H NMR (500 MHz, DMSO-d_6): δ = 7.46 (1H, t, $^{3}J_{HH}$ = 8.5 Hz, CH^2 of coumarin), 7.47 (1H, d, $^{3}J_{HH}$ = 8.3 Hz, CH^4 of coumarin), 7.63-7.66 (4H, m, 4CH of Ph), 7.77 (2H, t, $^{3}J_{HH}$ = 7.2 Hz, $2CH_{para}$ of Ph), 7.83 (1H, t, $^{3}J_{HH}$ = 7.7 Hz, CH^2 of coumarin), 8.15 (2H, t, $^{3}J_{HH}$ = 7.2 Hz, $2CH_{ortho}$ of Ph), 8.17 (2H, t, $^{3}J_{HH}$ = 7.2 Hz, $2CH_{ortho}$ of Ph), 8.55 (1H, d, $^{3}J_{HH}$ = 7.8 Hz, CH^1 of coumarin), 8.62 (1H, s, CH^7). MS (EI, 70 eV): m/z (%) = 414 (M^+, 100), 384 (12), 356 (24), 327 (24), 251 (22), 206 (18), 105 (13), 77 (16).

2-Chloro-6-oxo-8,10-diphenyl-6*H*-pyrano[3',4':4,5]cyclopenta[1,2-*c*]chromene-11-carbonitrile (3b).

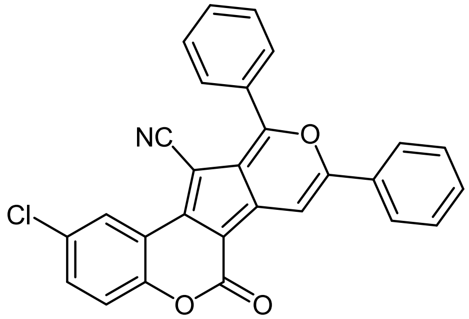

Orange powder, dec point = 318-320 °C, 0.37 g, yield: 84%. IR (KBr): 2202 (C≡N), 1730 (C=O), 1615, 1555, 1541, and 1468 (Ar), 1228, 1176, and 1005 (C-O) cm^{-1}. Anal. calcd. for

$C_{28}H_{14}ClNO_3$ (447.87): C, 75.09; H, 3.15, N, 3.13%. Found C, 75.07; H, 3.12, N, 3.12%. ^{1}H NMR (300 MHz, $CDCl_3$), δ = 7.35 (1H, d, $^3J_{HH}$ = 8.8 Hz, CH^2 of coumarin), 7.49 (1H, dd, $^3J_{HH}$ = 8.8 Hz, $^2J_{HH}$ = 2.5 Hz, CH^4 of coumarin), 7.56-7.60 (3H, m, 3CH of Ph), 7.73-7.76 (3H, m, 3CH of Ph), 8.07 (2H, t, $^3J_{HH}$ = 6.2 Hz, 2CH$_{ortho}$ of Ph), 8.09 (2H, t, $^3J_{HH}$ = 6.2 Hz, 2CH$_{ortho}$ of Ph), 8.66 (1H, d, $^3J_{HH}$ = 2.6 Hz, CH^1 of coumarin), 8.74 (1H, s, CH^7). MS (EI, 70 eV): *m/z* (%) = 447 (M$^+$, 100), 427 (3), 390 (6), 327 (13), 251 (9), 105 (11), 77 (14).

3-Methoxy-6-oxo-8,10-diphenyl-6*H*-pyrano[3',4':4,5]cyclopenta[1,2-*c*]chromene-11-carbonitrile (3c).

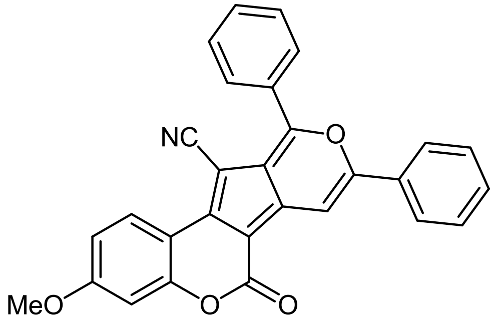

Orange powder, dec point = 343-345 °C, 0.34 g, yield: 77%. IR (KBr): 2198 (C≡N), 1727 (C=O), 1614, 1556, 1460, and 1421 (Ar), 1205, 1166, 1117, and 1035 (C-O) cm^{-1}. Anal. calcd. for $C_{29}H_{17}NO_4$ (443.45): C, 78.55; H, 3.86, N, 3.16%. Found: C, 78.54; H, 3.85, N, 3.14%. ^{1}H NMR (500 MHz, $CDCl_3$): δ = 3.92 (3H, s, OCH_3), 6.92 (1H, d, $^3J_{HH}$ = 2.5 Hz, CH^4 of coumarin), 6.95 (1H, dd, $^3J_{HH}$ = 8.5 Hz, $^2J_{HH}$ = 2.5 Hz, CH^2 of coumarin), 7.55-7.60 (3H, m, 3CH of Ph), 7.72-7.75 (3H, m, 3CH of Ph), 8.07 (4H, d, $^3J_{HH}$ = 8.7 Hz 4CH$_{ortho}$ of Ph), 8.66 (1H, d, $^3J_{HH}$ = 8.5 Hz, CH^1 of coumarin), 8.74 (1H, s, CH^7). MS (EI, 70 eV): *m/z* (%) = 443 (M$^+$, 100), 400 (20), 372 (10), 314 (18), 221 (23), 105 (38), 77 (24).

8-(2-Chlorophenyl)-6-oxo-10-phenyl-6*H*-pyrano[3',4':4,5]cyclopenta[1,2-*c*]chromene-11-carbonitrile (3d).

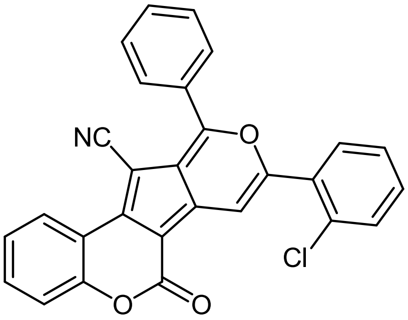

Orange powder, dec point = 255-257 °C, 0.38 g, yield: 86%. IR (KBr): 2204 (C≡N), 1706 (C=O), 1605, 1548, and 1468 (Ar), 1176, 1115, 1045, and 1029 (C-O) cm^{-1}. Anal. calcd. for $C_{28}H_{14}ClNO_3$ (447.87): C, 75.09; H, 3.15, N, 3.13%. Found: C, 75.06; H, 3.12, N, 3.12%. ^1H NMR (500 MHz, DMSO-d_6): δ = 7.42 (1H, t, $^3J_{HH}$ = 7.4 Hz, CH^2 of coumarin), 7.47 (1H, d, $^3J_{HH}$ = 8.0 Hz, CH^4 of coumarin), 7.59-7.66 (3H, m, 3CH of Ar), 7.70-7.74 (3H, m, 3CH of Ph), 7.78 (1H, t, $^3J_{HH}$ = 7.4 Hz, CH^3 of coumarin), 7.98 (1H, d, $^3J_{HH}$ = 7.0 Hz, CH of Ar), 8.11 (2H, d, $^3J_{HH}$ = 7.2 Hz, 2CH of Ar), 8.50 (1H, d, $^3J_{HH}$ = 7.8 Hz, CH^1 of coumarin), 8.51 (1H, s, CH^7). ^{13}C NMR (75 MHz, $CDCl_3$): δ = 80.76, 111.48, 117.00, 117.70, 117.94, 123.74, 124.72, 125.59, 127.49, 128.86, 129.77, 130.77, 131.01,131.09, 131.10, 131.47, 131.97, 132.20, 133.07, 139.31, 148.03, 153.31, 155.44, 158.49, 162.77. MS (EI, 70 eV): *m/z* (%) = 447 (M^+, 7), 308 (13), 251 (11), 139 (100), 105 (46), 77 (34), . Crystal data for 3d C28H14ClNO3 (CCDC 1970237): MW = 575.55, monoclinic, P 1 21/n 1, a = 7.4936(15) Å, b = 24.222(5) Å, c = 13.519(3) Å, α = 90, □ = 101.90(3), □ = 90, V = 2401.1(9) Å3, Z = 4, Dc = 1.474 mg/m3, F (000) = 1088, crystal dimension 0.50 □ 0.30 □ 0.20 mm, radiation, Mo K□ (□ = 0.71073 Å), 2.280 □ 2□ □ 24.499, intensity data were collected at 293(2) K with a Bruker APEX area-detector diffractometer, and employing □/2□ scanning technique, in the range of -8 □ h □ 8, 0 □ k □ 28, 0 □ l □ 15; the structure was solved by a direct method, all non-hydrogen atoms were positioned and anisotropic

thermal parameters refined from 3827 observed reflections with R (into) = 0.0286 by a full-matrix least-squares technique converged to R1 = 0.0695, and wR2 = 0.1724 [I>2sigma(I)].

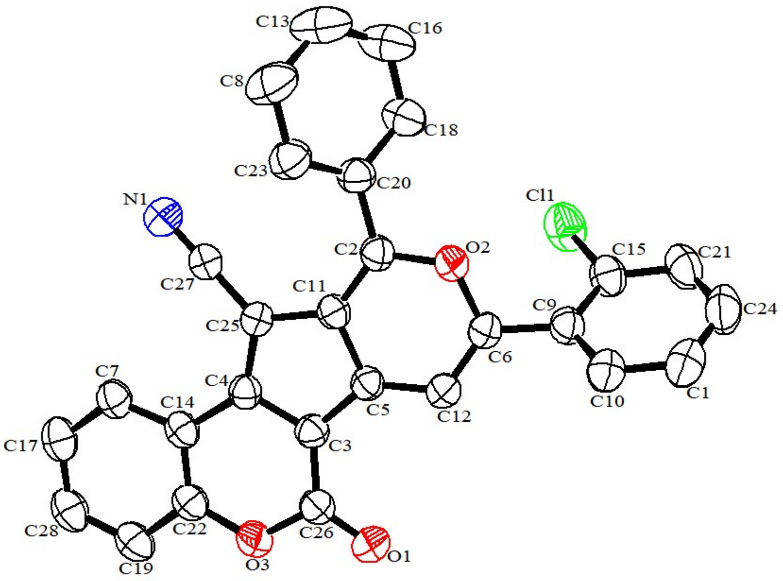

ORTEP diagram of **3d** (CCDC 1970237).

6-Oxo-8-phenyl-10-(*p*-tolyl)-6*H*-pyrano[3',4':4,5]cyclopenta[1,2-*c*]chromene-11-carbonitrile (3e).

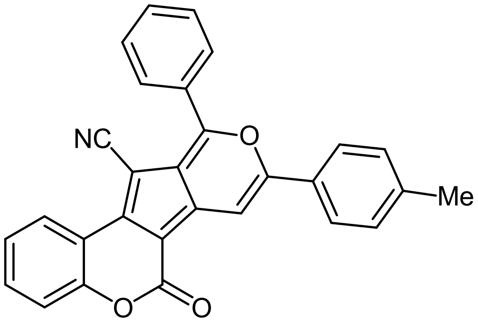

Orange powder, dec point = 342-345 °C, 0.32 g, yield: 75%. IR (KBr): 2198 (C≡N), 1730 (C=O), 1605, 1560, 1543, and 1511 (Ar), 1176, 1112, 1052, and 1036 (C-O) cm^{-1}. Anal. calcd. for $C_{29}H_{17}NO_3$ (427.45): C, 81.49; H, 4.01, N, 3.28%. Found: C, 81.44; H, 4.02, N, 3.26%. ^{1}H NMR (300 MHz, DMSO-d_6): δ = 2.41 (3H, s, CH_3), 7.44 (2H, d, $^{3}J_{HH}$ = 8.0 Hz, 2CH of Ar), 7.45 (1H,

t, $^3J_{HH}$ = 7.8 Hz, CH^2 of coumarin), 7.49 (1H, d, $^3J_{HH}$ = 7.8 Hz, CH^4 of coumarin), 7.67 (1H, t, $^3J_{HH}$ = 7.4 Hz, CH^3 of coumarin), 7.74 (1H, t, $^3J_{HH}$ = 8.5 Hz, CH$_{para}$ of Ph), 7.77 (2H, t, $^3J_{HH}$ = 8.5 Hz, 2CH$_{meta}$ of Ph), 8.05 (2H, d, $^3J_{HH}$ = 8.0 Hz, 2CH of Ar), 8.15 (2H, d, $^3J_{HH}$ = 8.4 Hz, 2CH$_{ortho}$ of Ph), 8.58 (1H, d, $^3J_{HH}$ = 7.7 Hz, CH^1 of coumarin), 8.62 (1H, s, CH^7). MS (EI, 70 eV): *m/z* (%) = 427 (M$^+$, 100), 370 (6), 354 (8), 340 (10), 327 (14), 264 (12), 105 (55), 77 (21).

8-(4-Nitrophenyl)-6-oxo-10-phenyl-6*H*-pyrano[3',4':4,5]cyclopenta[1,2-*c*]chromene-11-carbonitrile (3f).

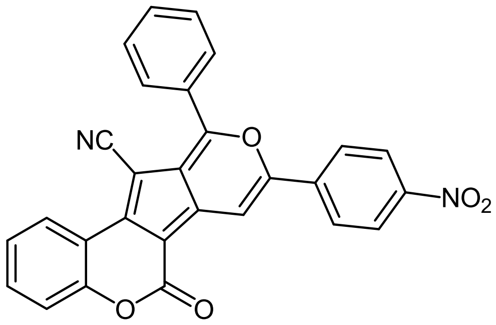

Orange powder, dec point = 367-369 °C, 0.41 g, yield: 91%. IR (KBr): 2204 (C≡N), 1720 (C=O), 1594, 1542, 1521, and 1424 (Ar), 1175, 1111, 1051, and 1000 (C-O) cm^{-1}. Anal. calcd. for $C_{28}H_{14}N_2O_5$ (458.09): C, 73.36; H, 3.08, N, 6.11%. Found: C, 73.34; H, 3.10, N, 6.13%. ^{1}H NMR (500 MHz, DMSO-d_6): δ = 7.43 (1H, t, $^3J_{HH}$ = 8.4 Hz, CH^2 of coumarin), 7.46 (1H, d, $^3J_{HH}$ = 8.6 Hz, CH^4 of coumarin), 7.63 (1H, t, $^3J_{HH}$ = 8.0 Hz, CH^3 of coumarin), 7.76 (2H, t , $^3J_{HH}$ = 7.6 Hz, 2CH$_{meta}$ of Ph), 7.82 (1H, t, $^3J_{HH}$ = 7.4 Hz, CH$_{para}$ of Ph), 8.17 (2H, d, $^3J_{HH}$ = 7.6 Hz, 2CH$_{ortho}$ of Ph), 8.38 (2H, d, $^3J_{HH}$ = 8.6 Hz, 2CH of Ar), 8.41 (2H, d, $^3J_{HH}$ = 8.6 Hz, 2CH of Ar), 8.55 (1H, d, $^3J_{HH}$ = 7.9 Hz, CH^1 of coumarin), 8.80 (1H, s, CH^7). MS (EI, 70 eV): *m/z* (%) = 458 (M$^+$, 100), 412 (23), 354 (30), 327 (62), 251 (28), 105 (71), 77 (46).

6-Oxo-8,10-di-*p*-tolyl-6*H*-pyrano[3',4':4,5]cyclopenta[1,2-*c*]chromene-11-carbonitrile (3g).

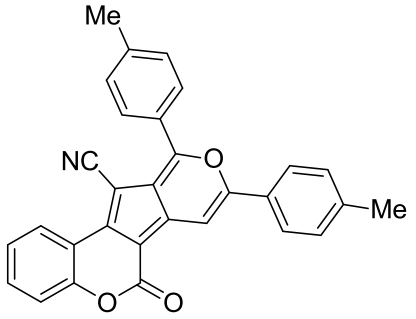

Orange powder, dec point = 348-350 °C, 0.31 g, yield: 72%. IR (KBr): 2198 (C≡N), 1730 (C=O), 1604, 1563, 1484, and 1422 (Ar), 1175, 1112, 1051, and 998 (C-O) cm^{-1}. Anal. calcd. for $C_{30}H_{19}NO_3$ (441.14): C, 81.62; H, 4.34, N, 3.17%. Found: C, 81.63; H, 4.36, N, 3.15%. ^1H NMR (500 MHz, DMSO-d_6): δ = 2.47 (3H, s, CH_3), 2.55 (3H, s, CH_3), 7.37 (2H, d, $^3J_{HH}$ = 7.7 Hz, 2CH of Ar), 7.38 (1H, t, $^3J_{HH}$ = 7.2 Hz, CH^2 of coumarin), 7.43 (1H, d, $^3J_{HH}$ = 8.2 Hz, CH^4 of coumarin), 7.53 (2H, d, $^3J_{HH}$ = 7.7 Hz, 2CH of Ar), 7.56 (1H, t, $^3J_{HH}$ = 7.7 Hz, CH^3 of coumarin), 7.96 (2H, d, $^3J_{HH}$ = 7.8 Hz, 2CH of Ar), 7.99 (2H, d, $^3J_{HH}$ = 7.9 Hz, 2CH of Ar), 8.72 (1H, s, CH^7), 8.77 (1H, d, $^3J_{HH}$ = 7.9 Hz, CH^1 of coumarin). MS (EI, 70 eV): *m/z* (%) = 441 (M^+, 100), 354 (16), 264 (19), 220 (29), 119 (25), 91 (57).

10-(4-Bromophenyl)-6-oxo-8-phenyl-6*H*-pyrano[3',4':4,5]cyclopenta[1,2-*c*]chromene-11-carbonitrile (3h).

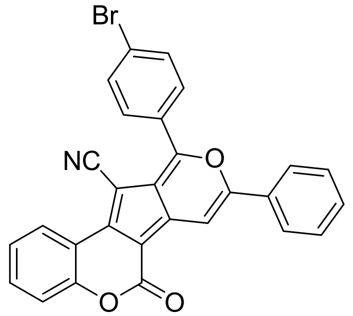

Orange powder, dec point = 357-359 °C, 0.40 g, yield: 83%. IR (KBr): 2204 (C≡N), 1729 (C=O), 1602, 1563, 1541, and 1425 (Ar), 1175, 1113, 1051, and 1005 (C-O) cm^{-1}. Anal. calcd. for

$C_{28}H_{14}BrNO_3$ (491.02): C, 68.31; H, 2.87, N, 2.85%. Found: C, 68.33; H, 2.85, N, 2.86%. ^{1}H NMR (500 MHz, DMSO-d_6): δ = 7.48 (1H, t, $^3J_{HH}$ = 8.2 Hz, CH^2 of coumarin), 7.49 (1H, d, $^3J_{HH}$ = 8.4 Hz, CH^4 of coumarin), 7.63 (2H, d, $^3J_{HH}$ = 7.0 Hz, 2CH of Ar), 7.64 (1H, t, $^3J_{HH}$ = 8.2 Hz, CH$_{para}$ of Ph), 7.67 (1H, t, 7.2 Hz, CH^3 of coumarin), 7.98 (2H, d, $^3J_{HH}$ = 8.2 Hz, 2CH$_{meta}$ of Ph), 8.11 (2H, d, $^3J_{HH}$ = 8.2 Hz, 2CH$_{ortho}$ of Ph), 8.16 (2H, d, $^3J_{HH}$ = 7.0 Hz, 2CH of Ar), 8.56 (1H, d, $^3J_{HH}$ = 7.0 Hz, CH^1 of coumarin), 8.66 (1H, s, CH^7). MS (EI, 70 eV): *m/z* (%) = 493 (M^++1, 100), 491 (M^+, 98), 327 (26), 251 (30), 105 (27), 77 (41).

10-(4-Chlorophenyl)-6-oxo-8-phenyl-6*H*-pyrano[3',4':4,5]cyclopenta[1,2-*c*]chromene-11-carbonitrile (3i).

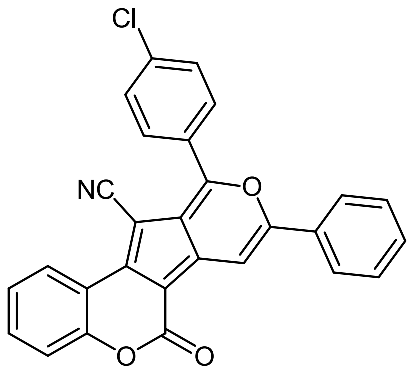

Orange powder, dec point = 370-373 °C, 0.36 g, yield: 81%. IR (KBr): 2200 (C≡N), 1729 (C=O), 1602, 1563, 1468, and 1423 (Ar), 1225, 1175, 1112, and 1051 (C-O) cm^{-1}. Anal. calcd. for $C_{28}H_{14}ClNO_3$ (447.87): C, 75.09; H, 3.15, N, 3.13%. Found: C, 75.06; H, 3.13, N, 3.12%. ^{1}H NMR (500 MHz, $CDCl_3$): δ = 7.42 (1H, t, $^3J_{HH}$ = 7.3 Hz, CH^2 of coumarin), 7.46 (1H, d, $^3J_{HH}$ = 8.2 Hz, CH^4 of coumarin), 7.50-7.64 (4H, m, 2CH$_{meta}$ of Ph, CH$_{para}$ of Ph, and CH^3 of coumarin), 7.74 (2H, d, $^3J_{HH}$ = 8.5 Hz, 2CH of Ar), 8.06 (2H, d, $^3J_{HH}$ = 8.5 Hz, 2CH of Ar), 8.09 (2H, d, $^3J_{HH}$ = 8.0 Hz, 2CH$_{meta}$ of Ph), 8.78 (1H, d, $^3J_{HH}$ = 7.9 Hz, CH^1 of coumarin), 8.81 (1H, s, CH^7). MS (EI, 70 eV): *m/z* (%) = 447 (M^+, 2), 327 (10), 308 (9), 251 (18), 138 (100), 105 (31), 77 (28).

8-(4-Methoxyphenyl)-6-oxo-10-phenyl-6*H*-pyrano[3',4':4,5]cyclopenta[1,2-*c*]chromene-11-carbonitrile (3j).

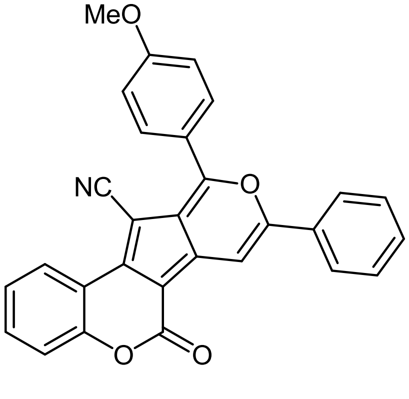

Orange powder, dec point = 318-320 °C, 0.35 g, yield: 80%. IR (KBr): 2198 (C≡N), 1735 (C=O), 1603, 1559, 1547, and 1470 (Ar), 1000 (C-O) cm^{-1}. Anal. calcd. for $C_{29}H_{17}NO_4$ (443.45): C, 78.55; H, 3.86, N, 3.16%. Found: C, 78.54; H, 3.84, N, 3.15%. ^{1}H NMR (500 MHz, DMSO-d_6): δ = 3.95 (3H, s, OCH_3), 7.28 (2H, d, $^3J_{HH}$ = 8.7 Hz, 2CH of Ar), 7.40 (1H, t, $^3J_{HH}$ = 7.1 Hz, CH^4 of coumarin), 7.41 (1H, d, $^3J_{HH}$ = 7.1 Hz, CH^2 of coumarin), 7.50-7.60 (3H, m, 3CH of Ph), 7.60 (1H, t, $^3J_{HH}$ = 6.8 Hz, CH^3 of coumarin), 8.07 (2H, d, $^3J_{HH}$ = 7.3 Hz, 2CH_{ortho} of Ph), 8.11 (2H, d, $^3J_{HH}$ = 8.6 Hz, 2CH of Ar), 8.48 (1H, s, CH^7), 8.51 (1H, d, $^3J_{HH}$ = 7.0 Hz, CH^1 of coumarin). MS (EI, 70 eV: *m/z* (%) = 443 (M^+, 100), 428 (2), 372 (7), 344 (7), 314 (9), 221 (16), 105 (20), 77 (13).

6-Oxo-8-phenyl-10-(*p*-tolyl)-6*H*-pyrano[3',4':4,5]cyclopenta[1,2-*c*]chromene-11-carbonitrile (3k).

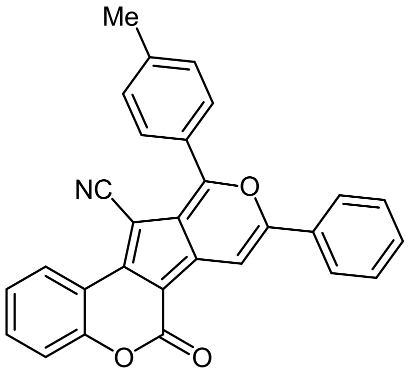

Orange powder, dec point = 301-305 °C, 0.33 g, yield: 78%. IR (KBr): 2181 (C≡N), 1680 (C=O), 1652, 1616, 1598, and 1486 (Ar), 1218, 1201, 1169, and 1105 (C-O) cm^{-1}. Anal. calcd. for $C_{29}H_{17}NO_3$ (427.12): C, 81.49; H, 4.01, N, 3.28%. Found: C, 81.51; H, 4.02, N, 3.28%. ^1H NMR (500 MHz, DMSO-d_6): δ = 2.56 (3H, s, CH_3), 7.38 (1H, t, $^3J_{HH}$ = 7.6 Hz, CH^2 of coumarin), 7.43 (1H, d, $^3J_{HH}$ = 8.3 Hz, CH^2 of coumarin), 7.52-7.60 (6H, m, 6CH of Ar), 8.00 (2H, d, $^3J_{HH}$ = 7.7 Hz, $2CH_{ortho}$ of Ph), 8.07 (2H, d, $^3J_{HH}$ = 7.5 Hz, 2CH of Ar), 8.77 (1H, s, CH^7), 8.78 (1H, d, $^3J_{HH}$ = 7.9 Hz, CH^1 of coumarin). MS (EI, 70 eV: m/z (%) = 427 (M^+, 100), 370 (4), 340 (5), 327 (4), 213 (9).

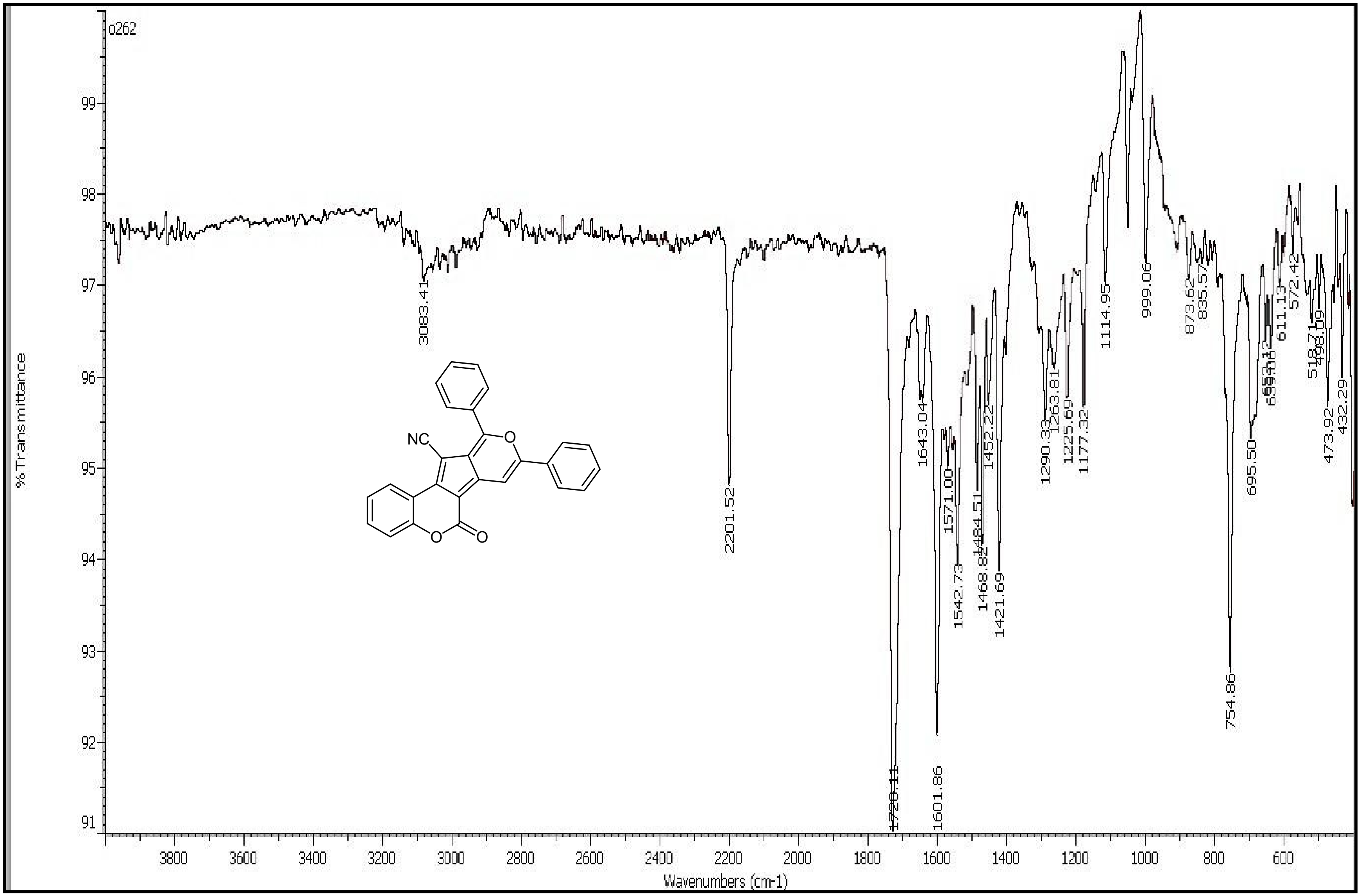

IR spectrum of **3a**

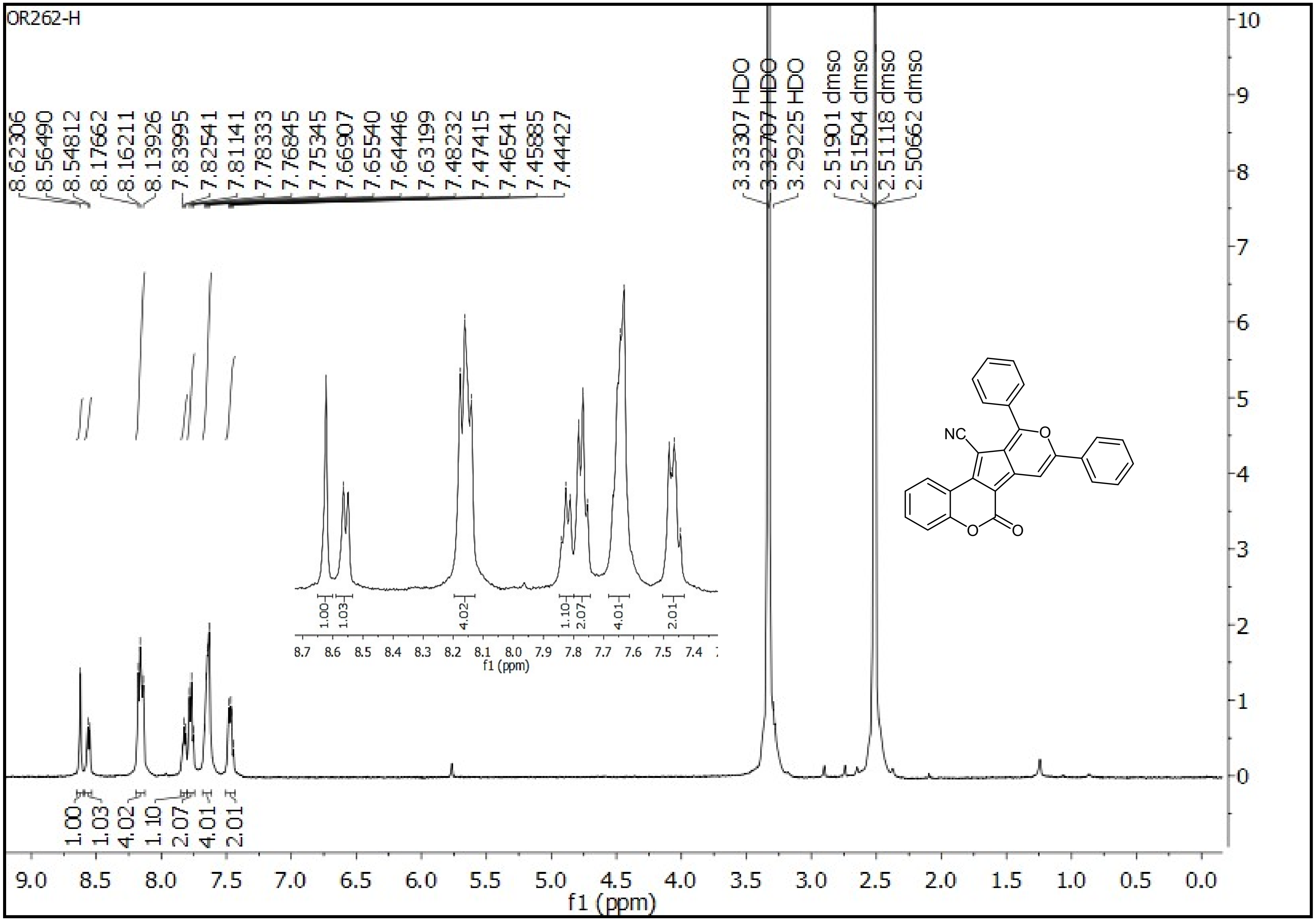

^{1}H NMR (DMSO-d_6, 500 MHz) of **3a**

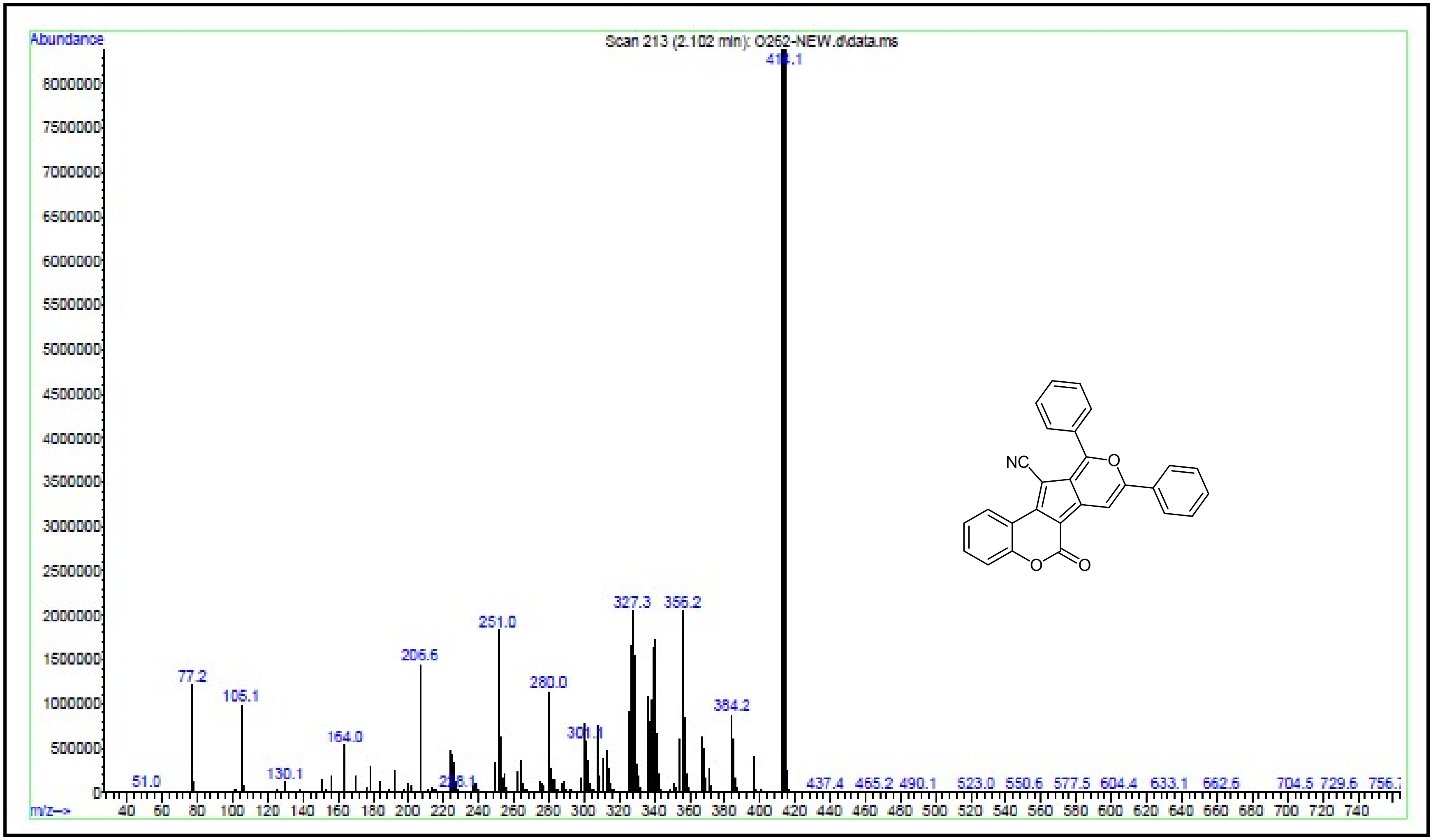

Mass spectrum of **3a**

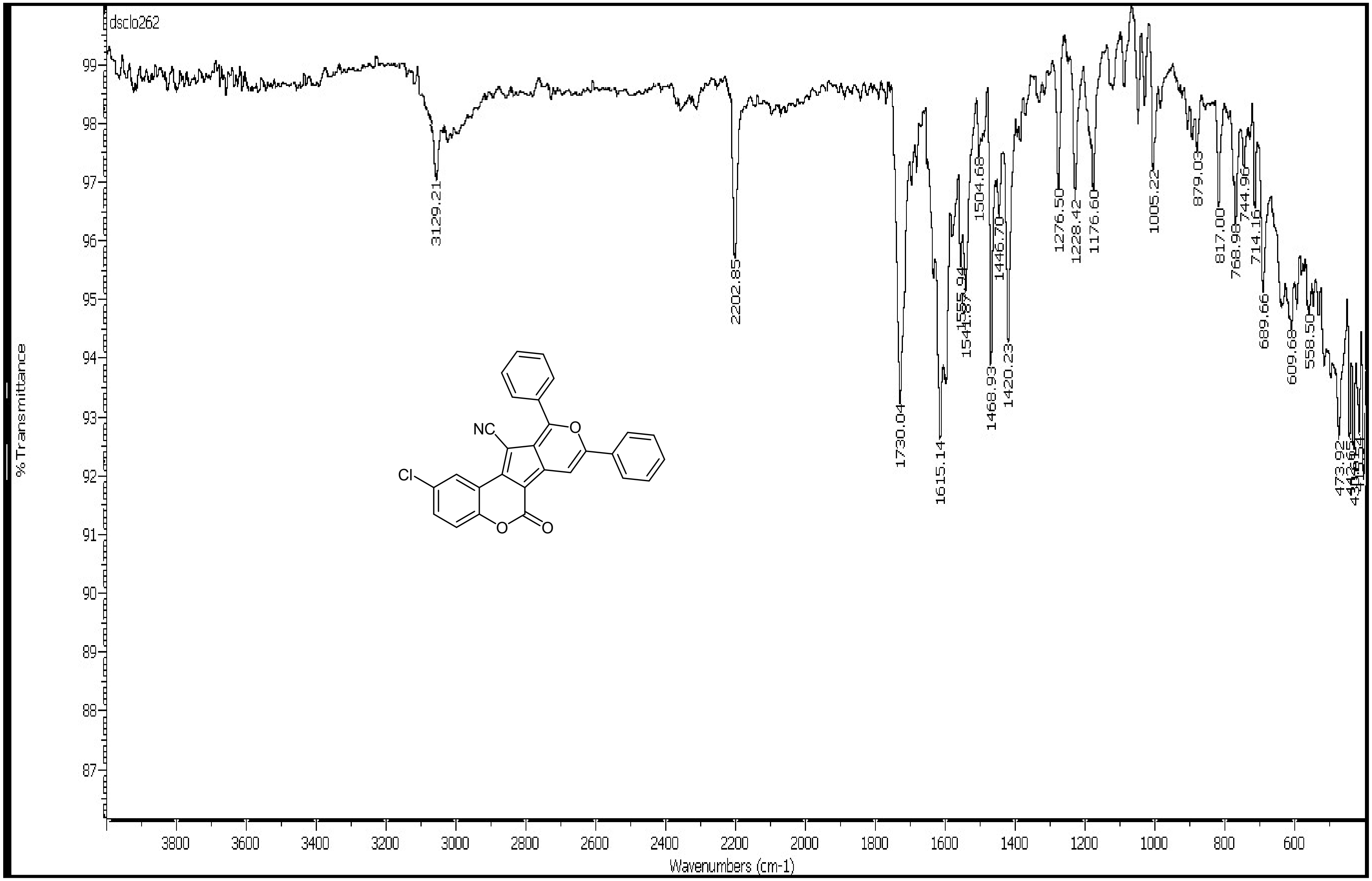

IR spectrum of **3b**

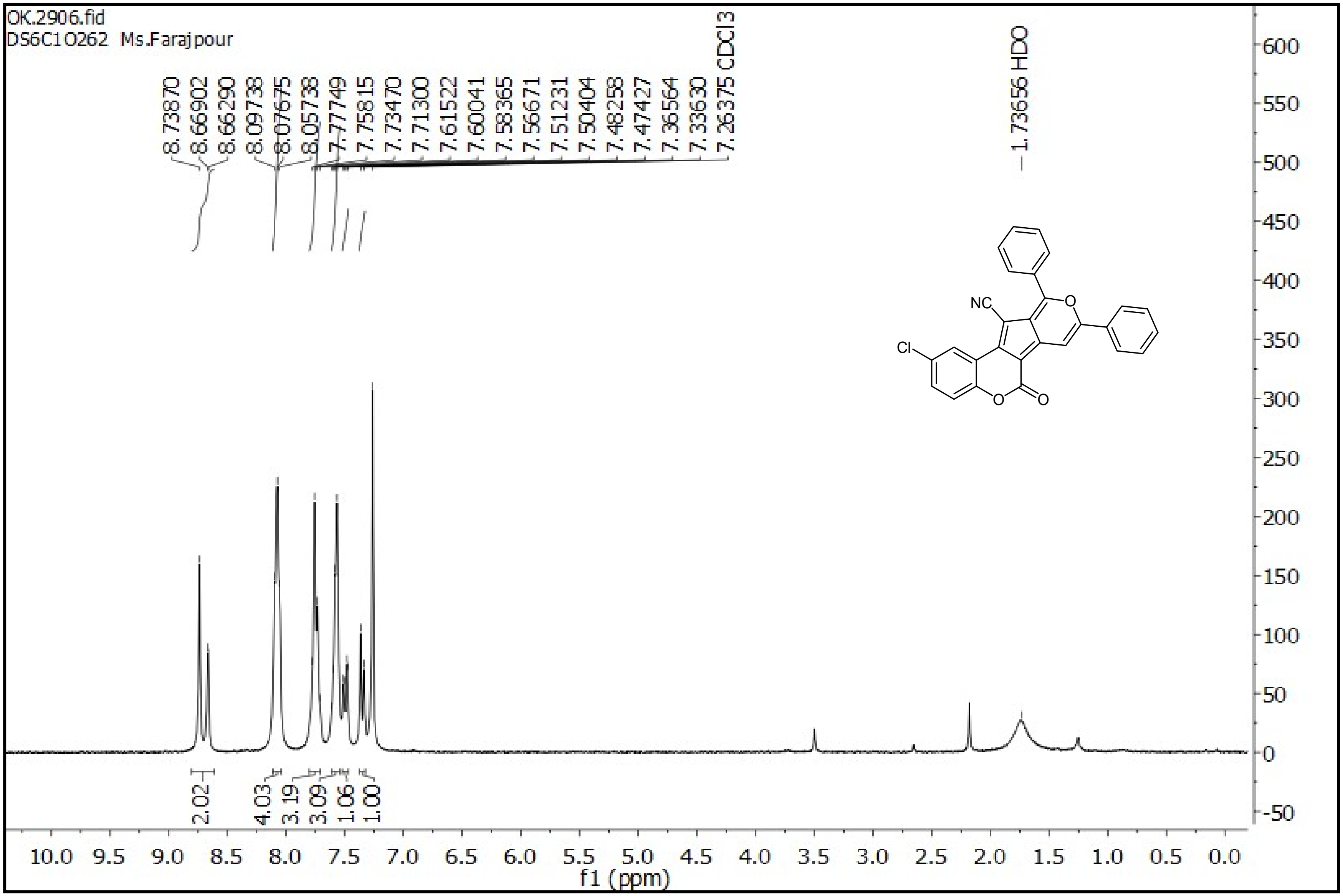

^{1}H NMR ($CDCl_3$, 300 MHz) of **3b**

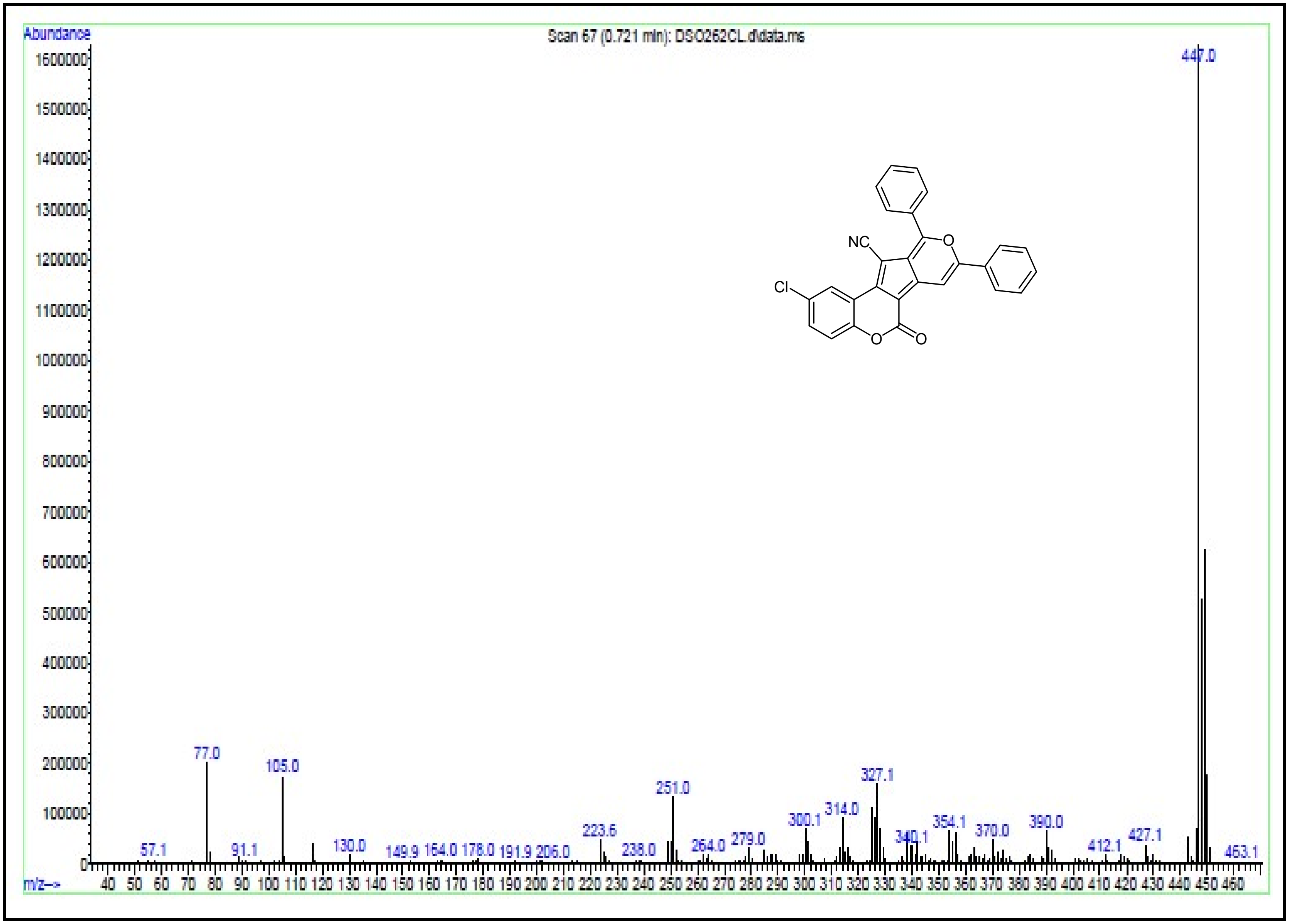

Mass spectrum of **3b**

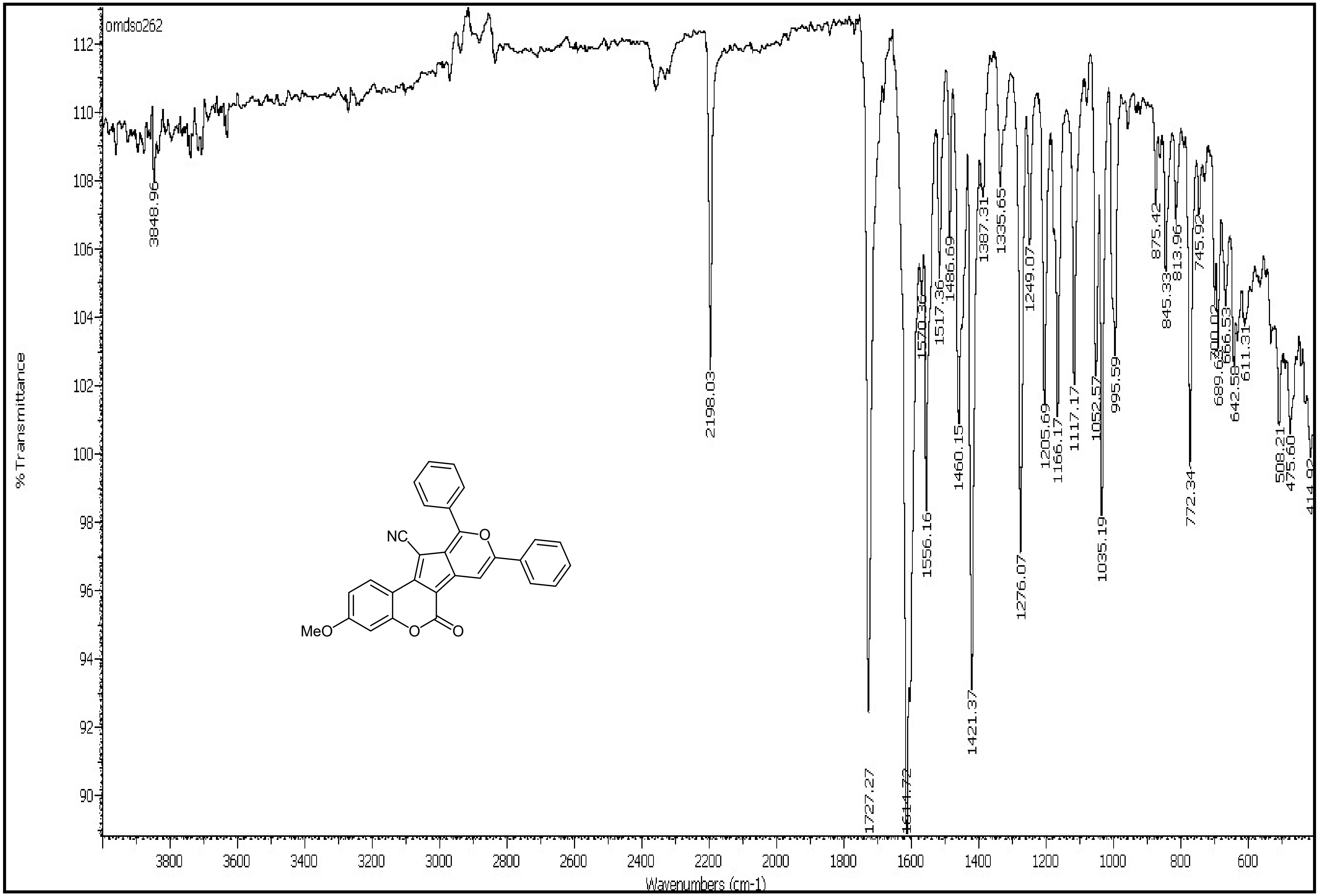

IR spectrum of **3c**

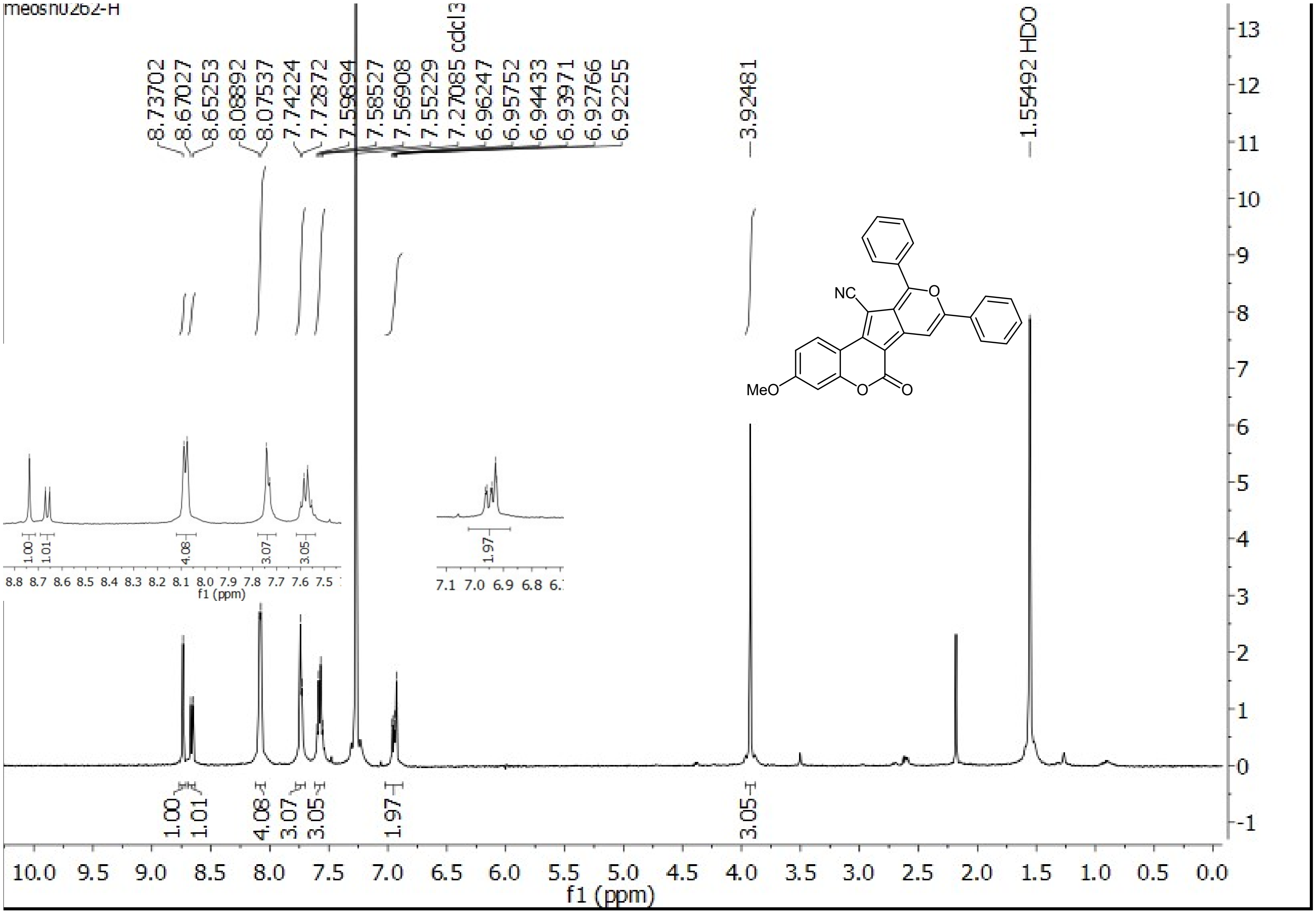

^{1}H NMR ($CDCl_3$, 500 MHz) of **3c**

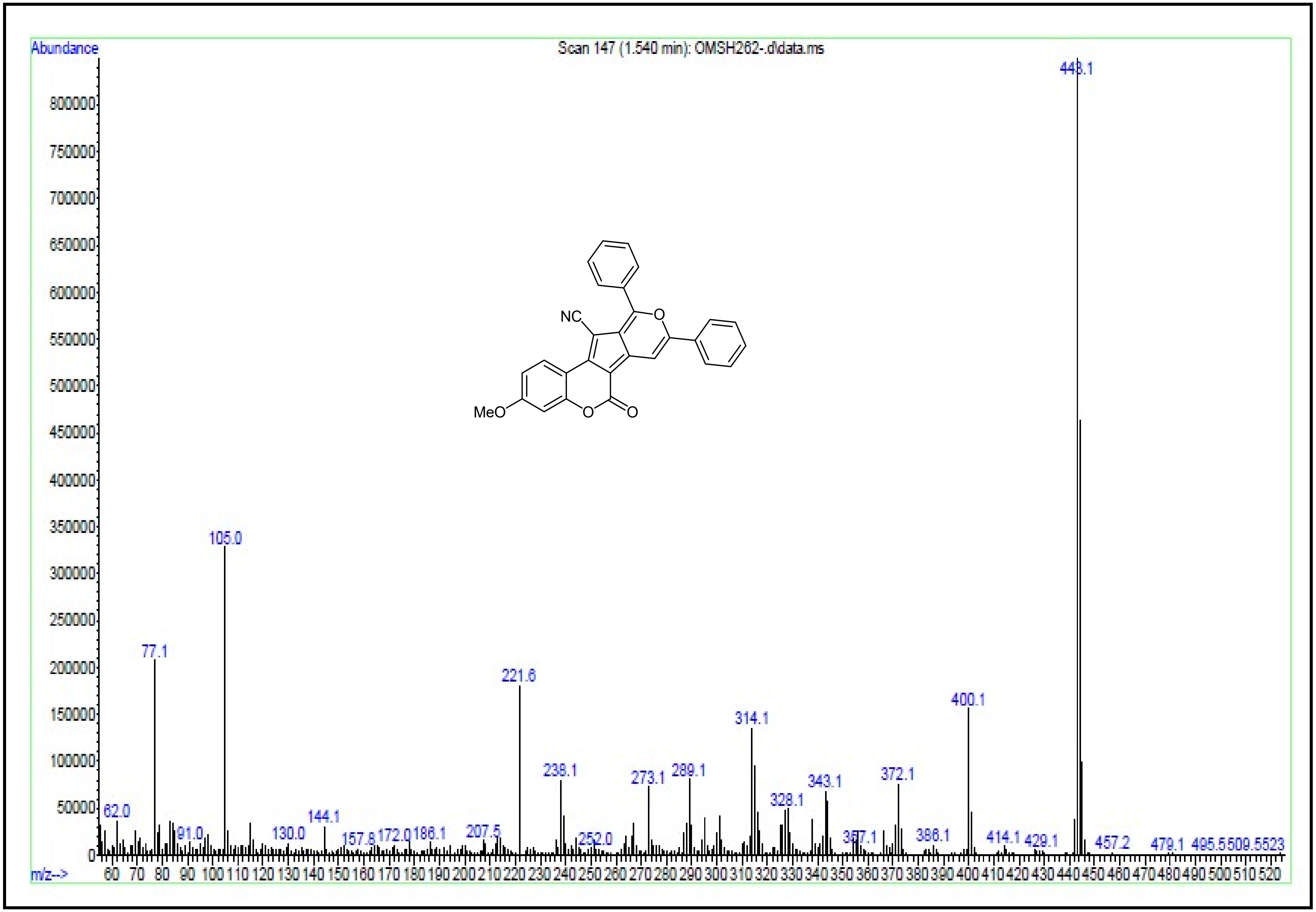

Mass spectrum of **3c**

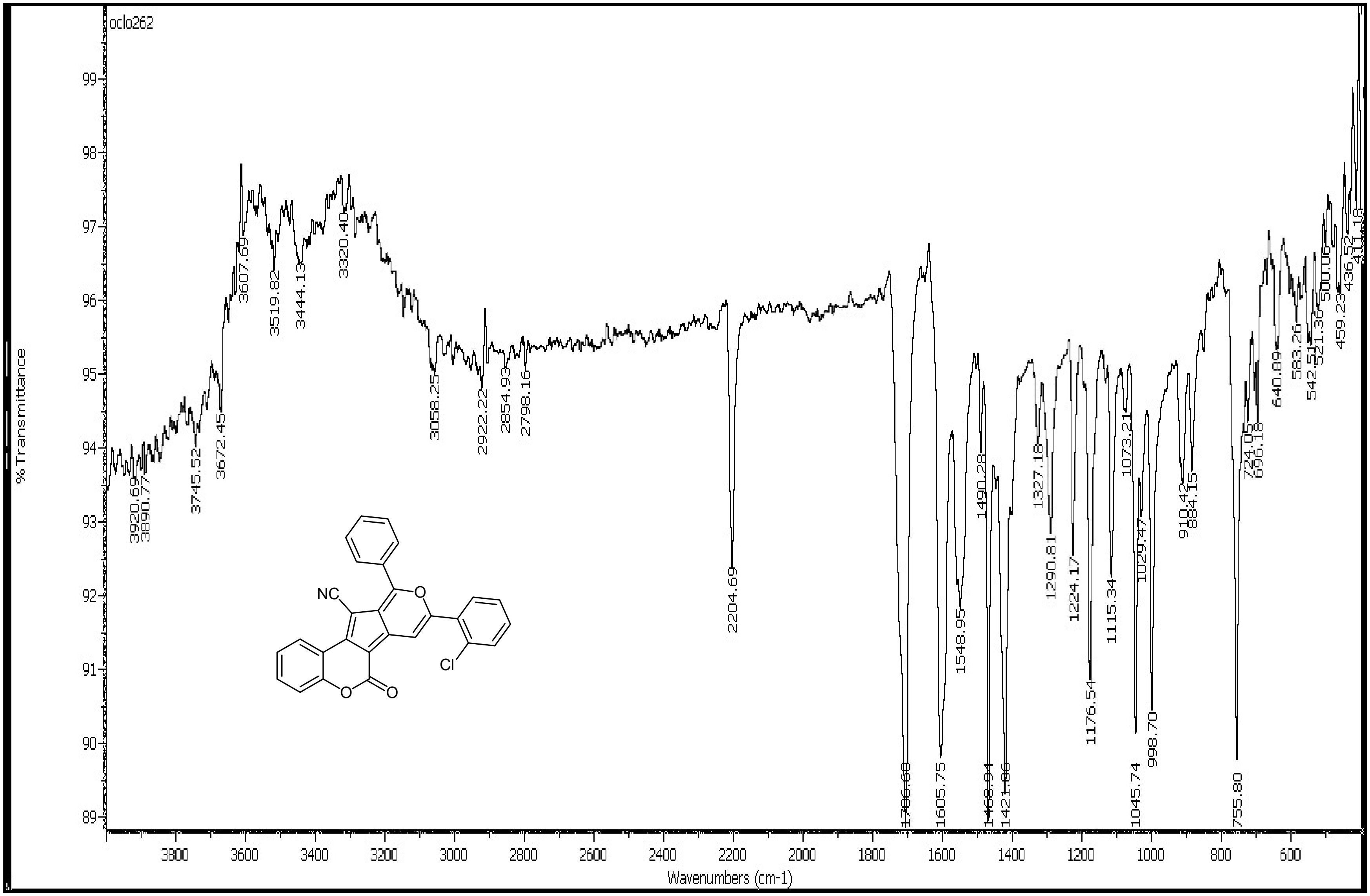

IR spectrum of **3d**

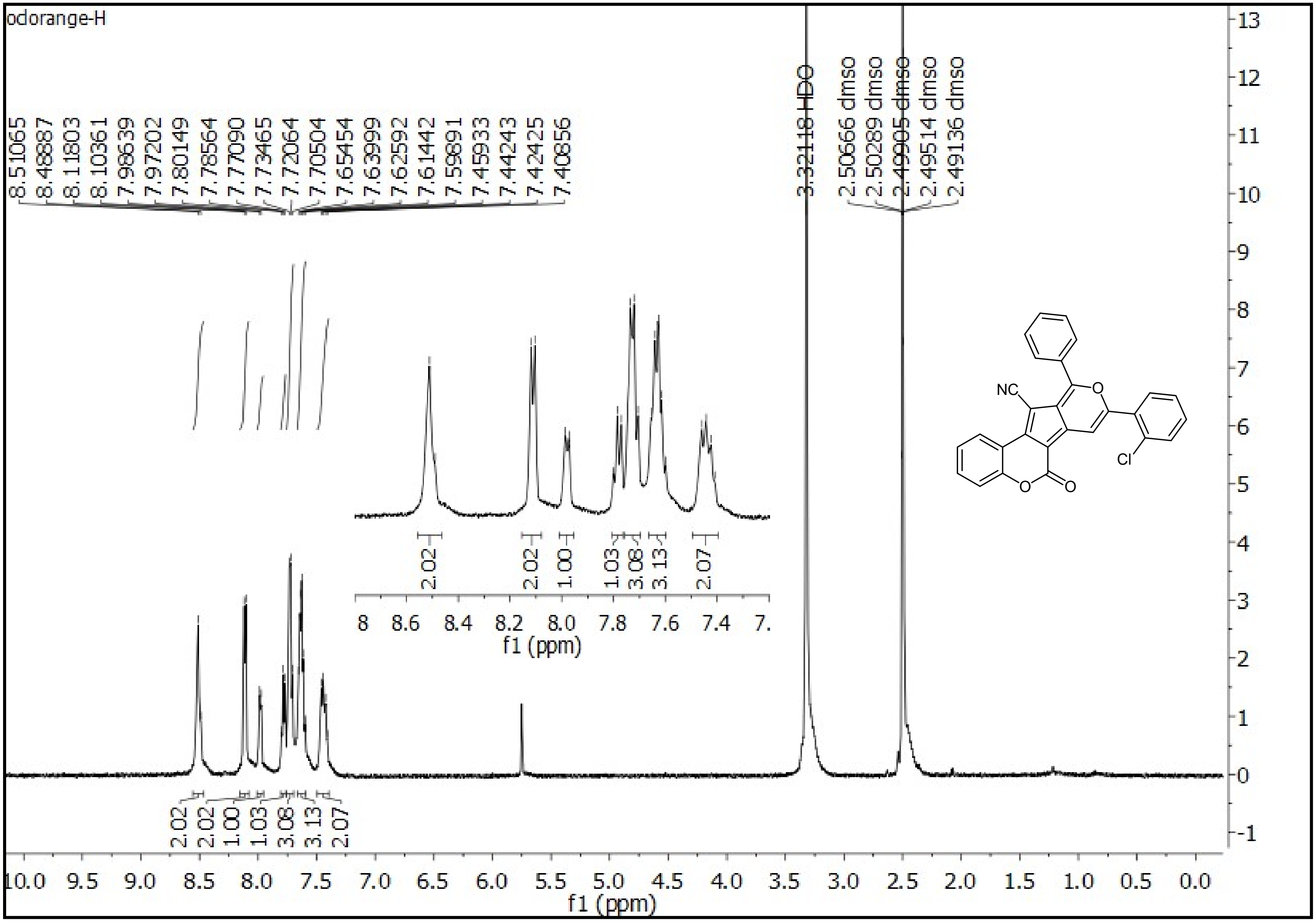

^{1}H NMR (DMSO-d_6, 500 MHz) of **3d**

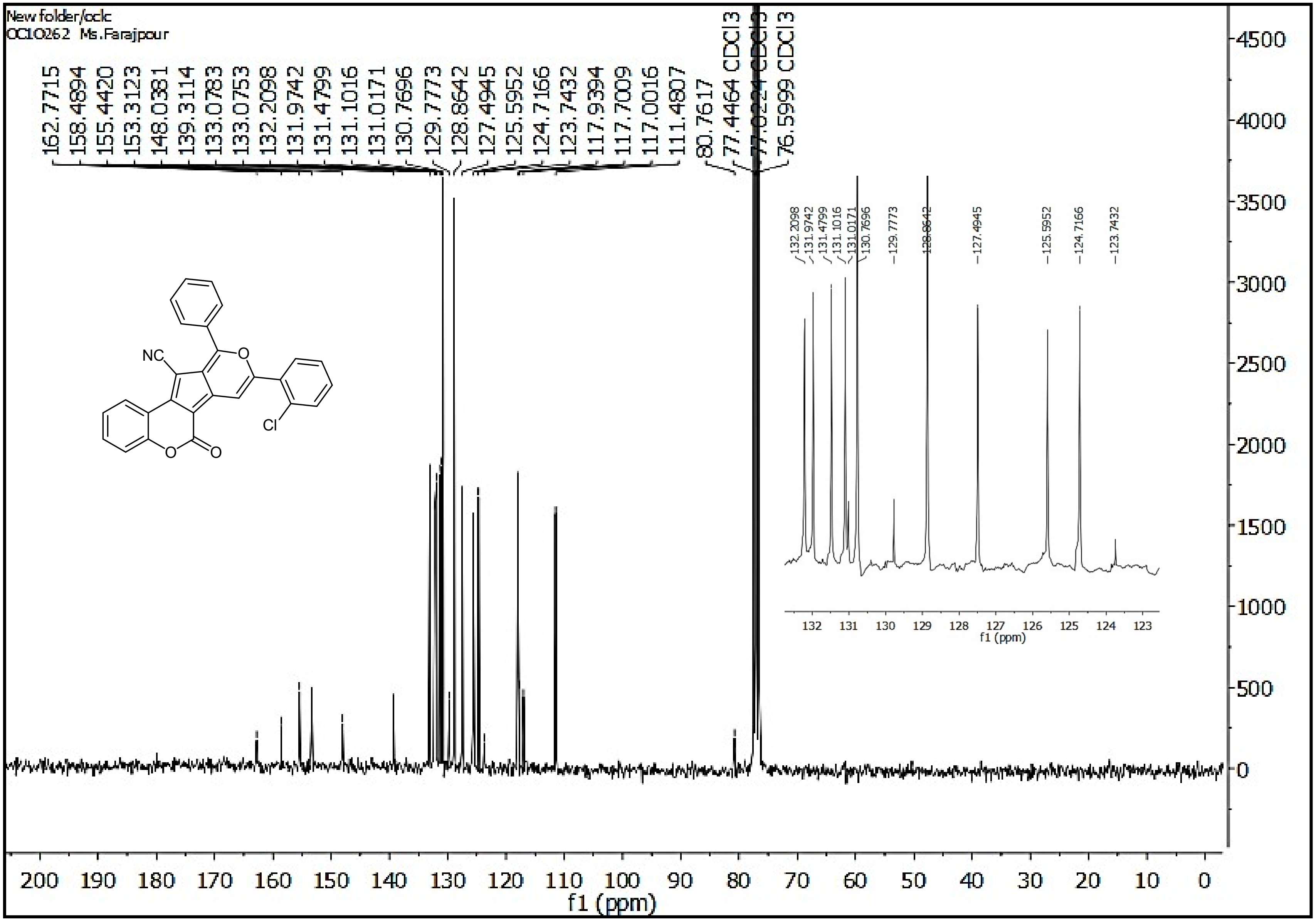

$^{13}C\{^{1}H\}$ NMR ($CDCl_3$, 75 MHz) of **3d**

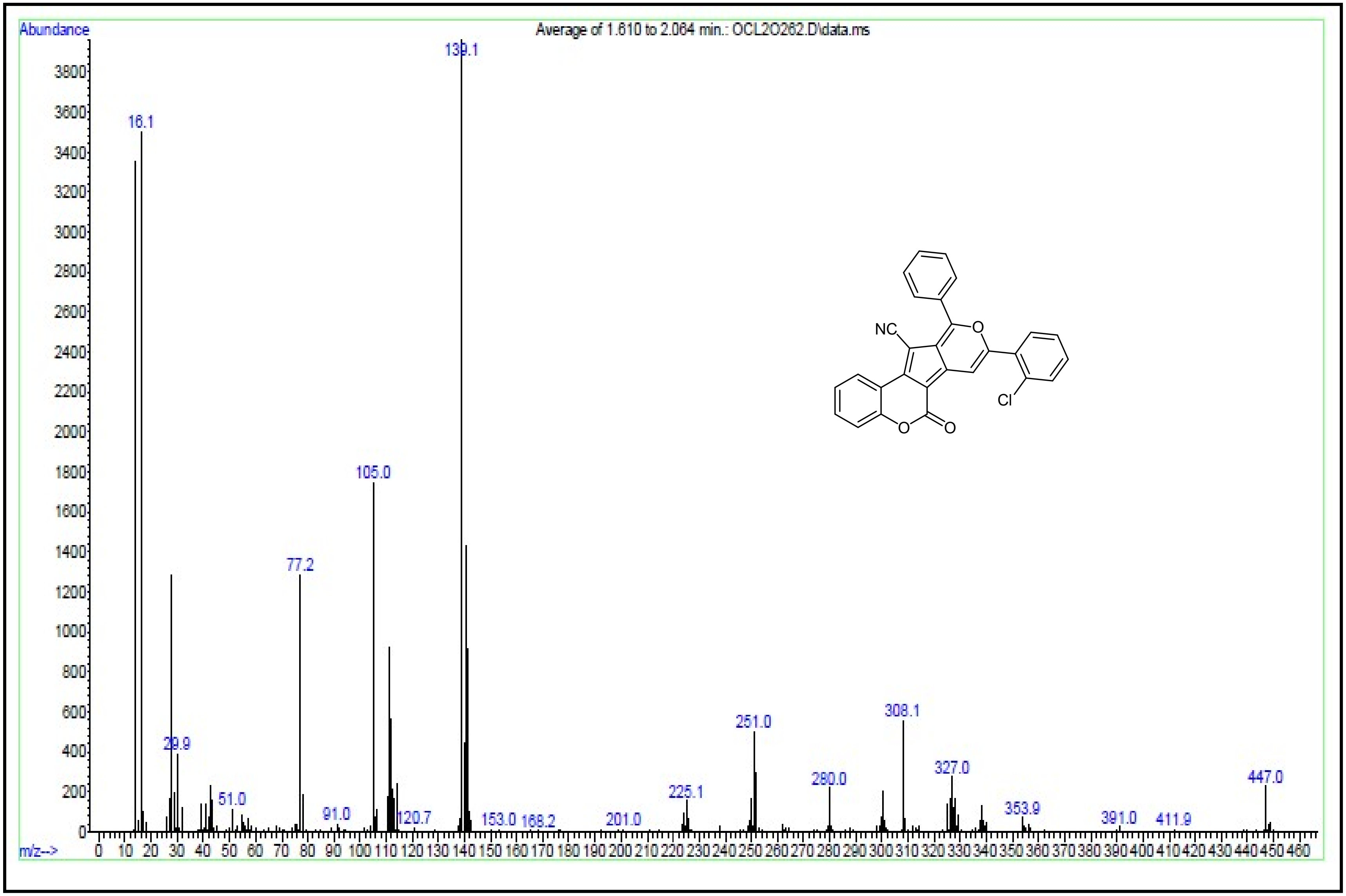

Mass spectrum of **3d**

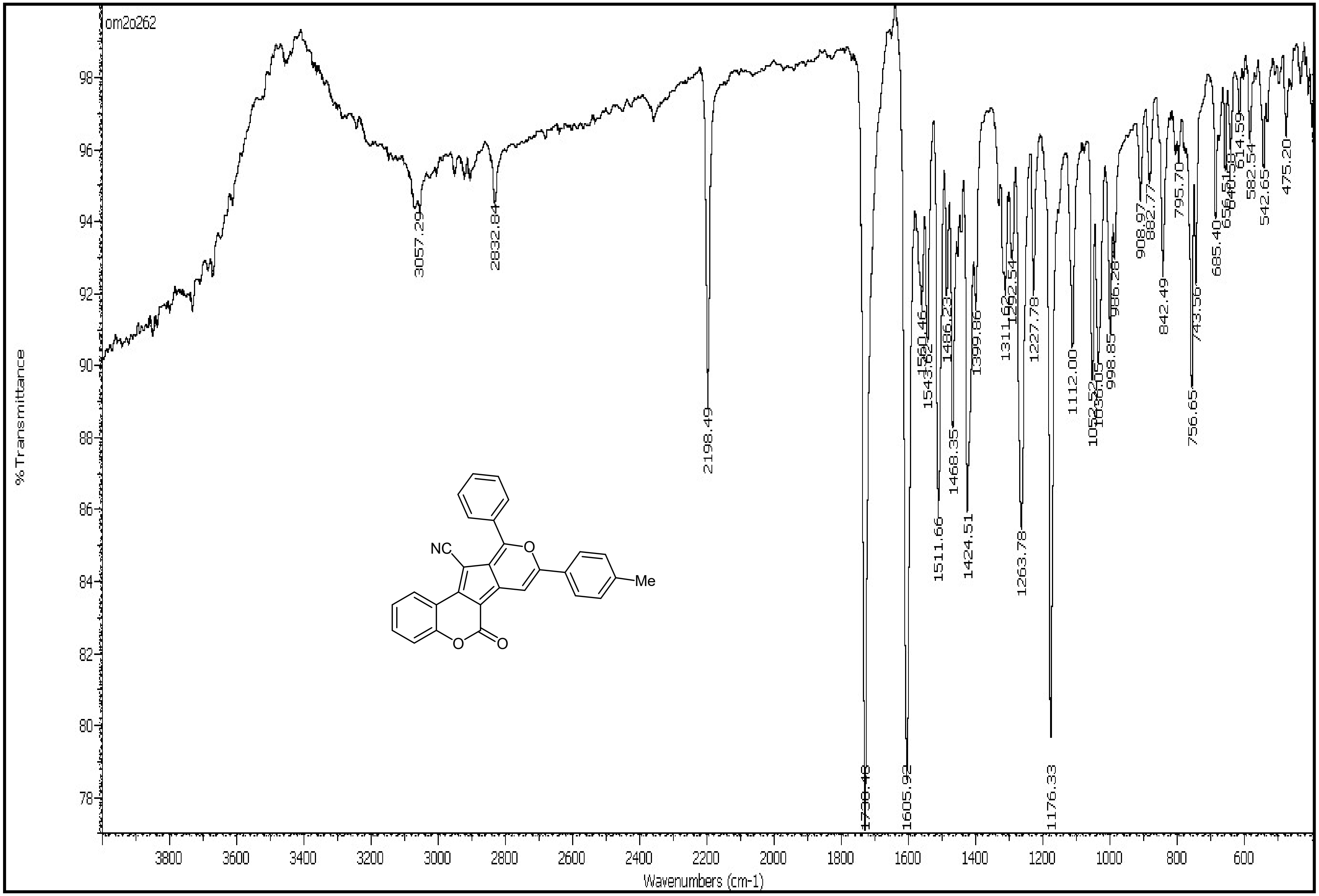

IR spectrum of **3e**

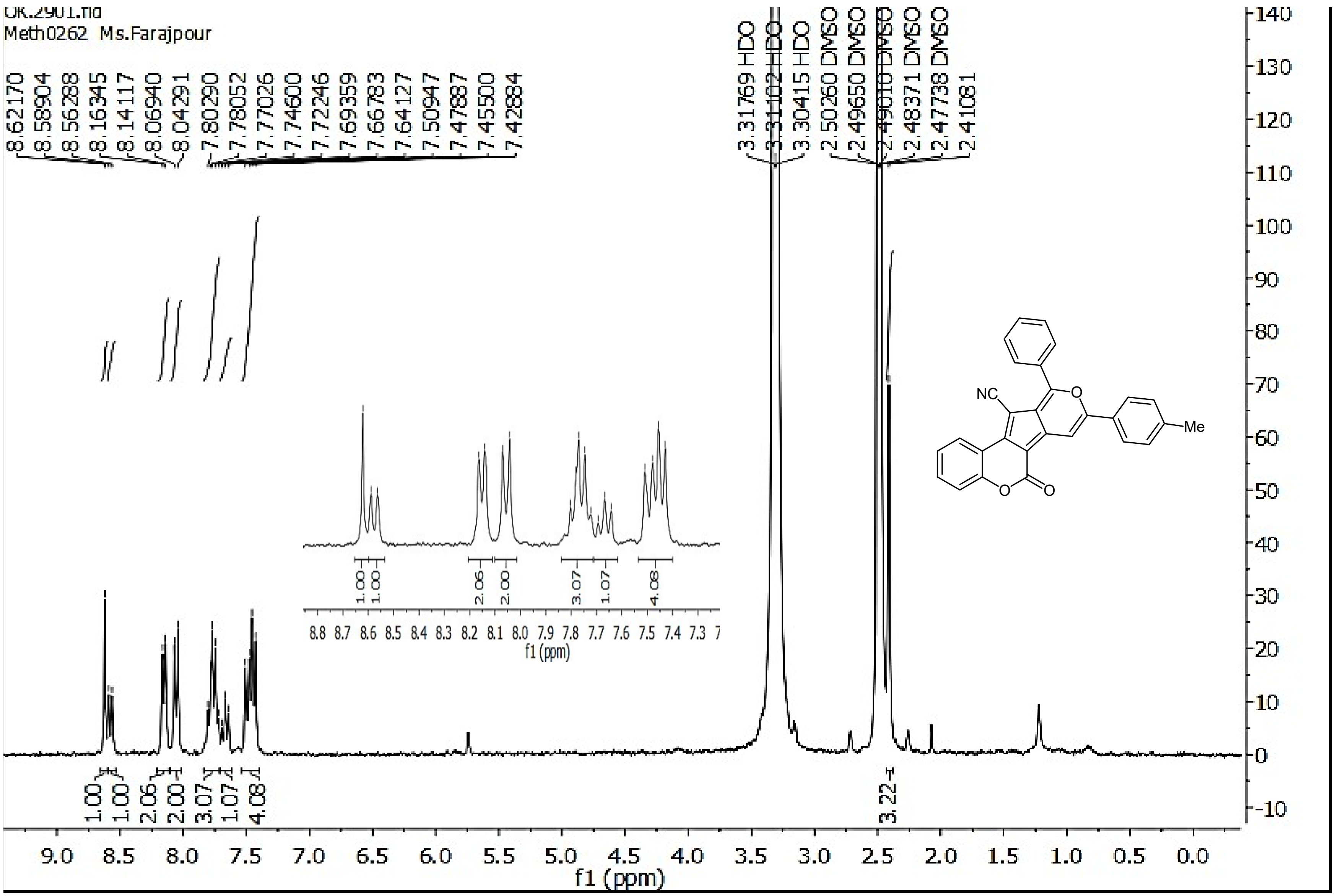

^{1}H NMR (DMSO-d_6, 300 MHz) of **3e**

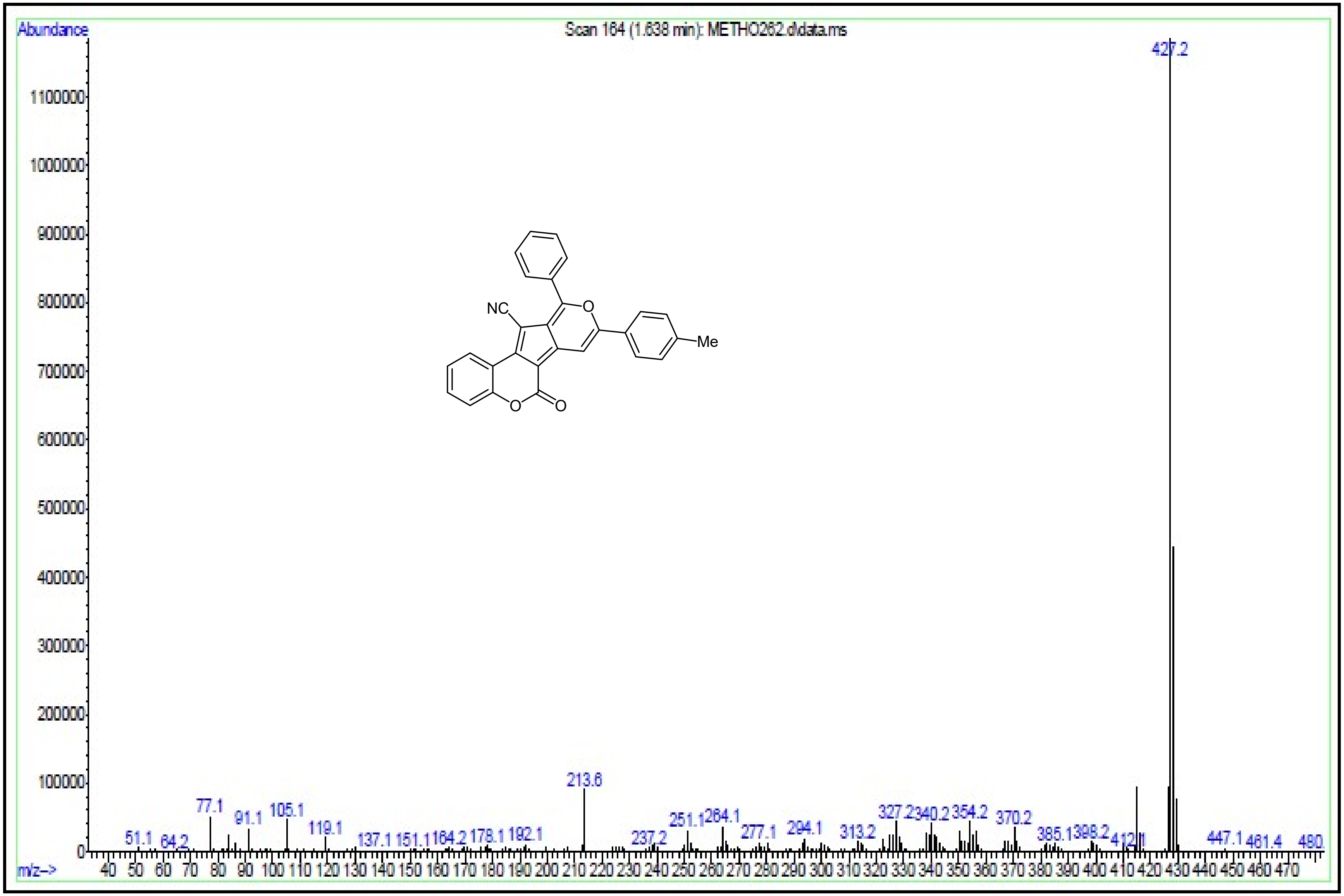

Mass spectrum of **3e**

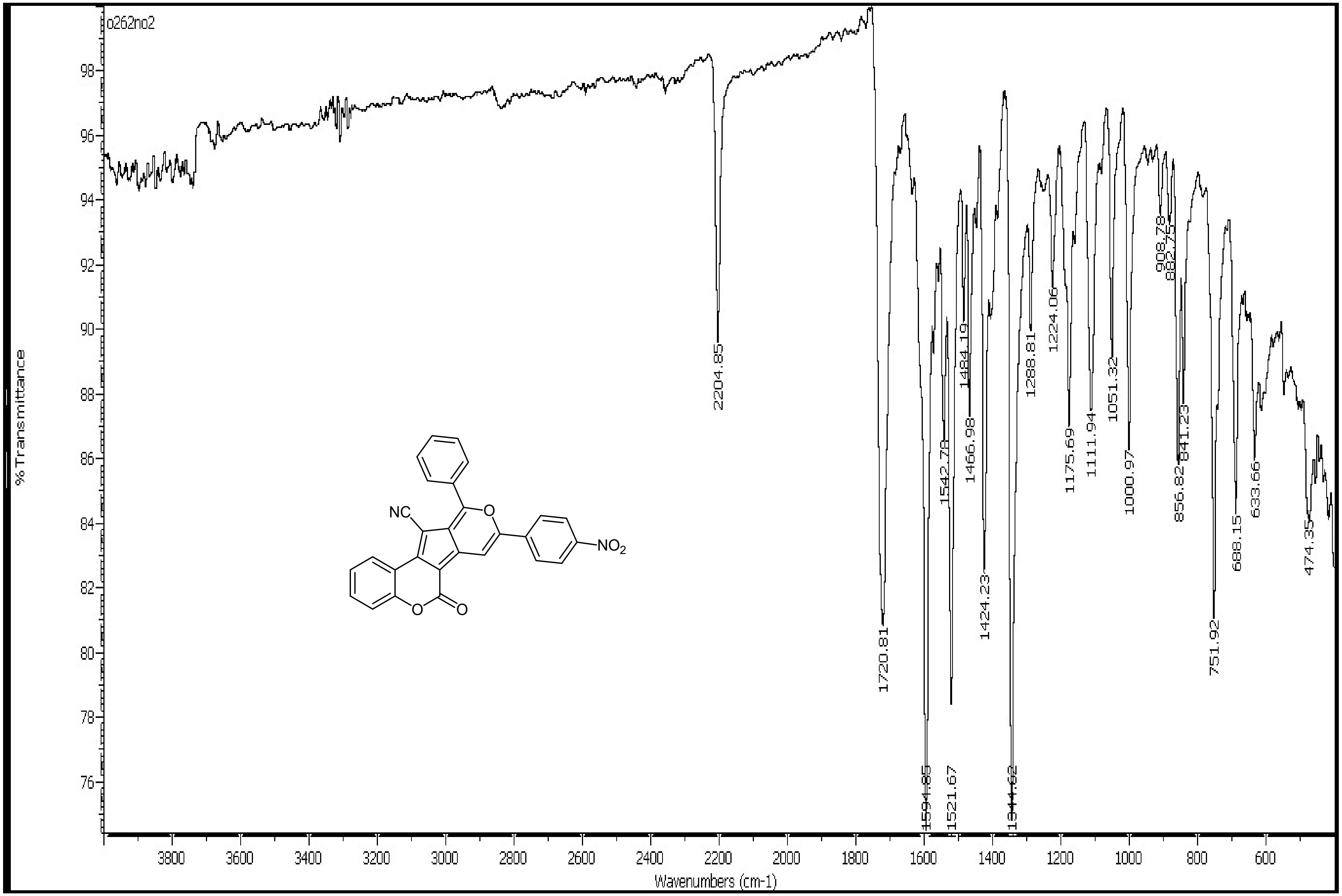

IR spectrum of **3f**

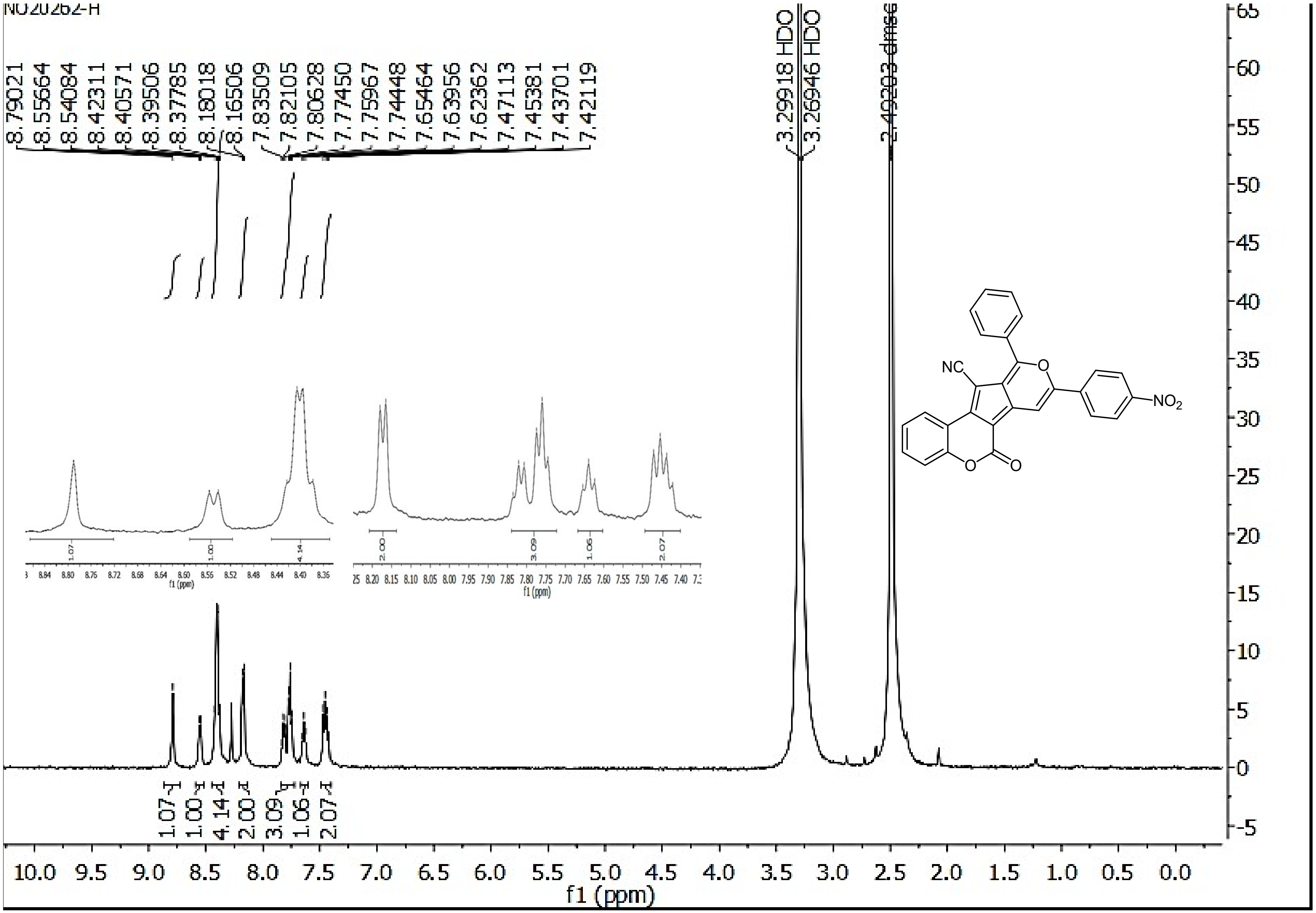

^{1}H NMR (DMSO-d_6, 300 MHz) of **3f**

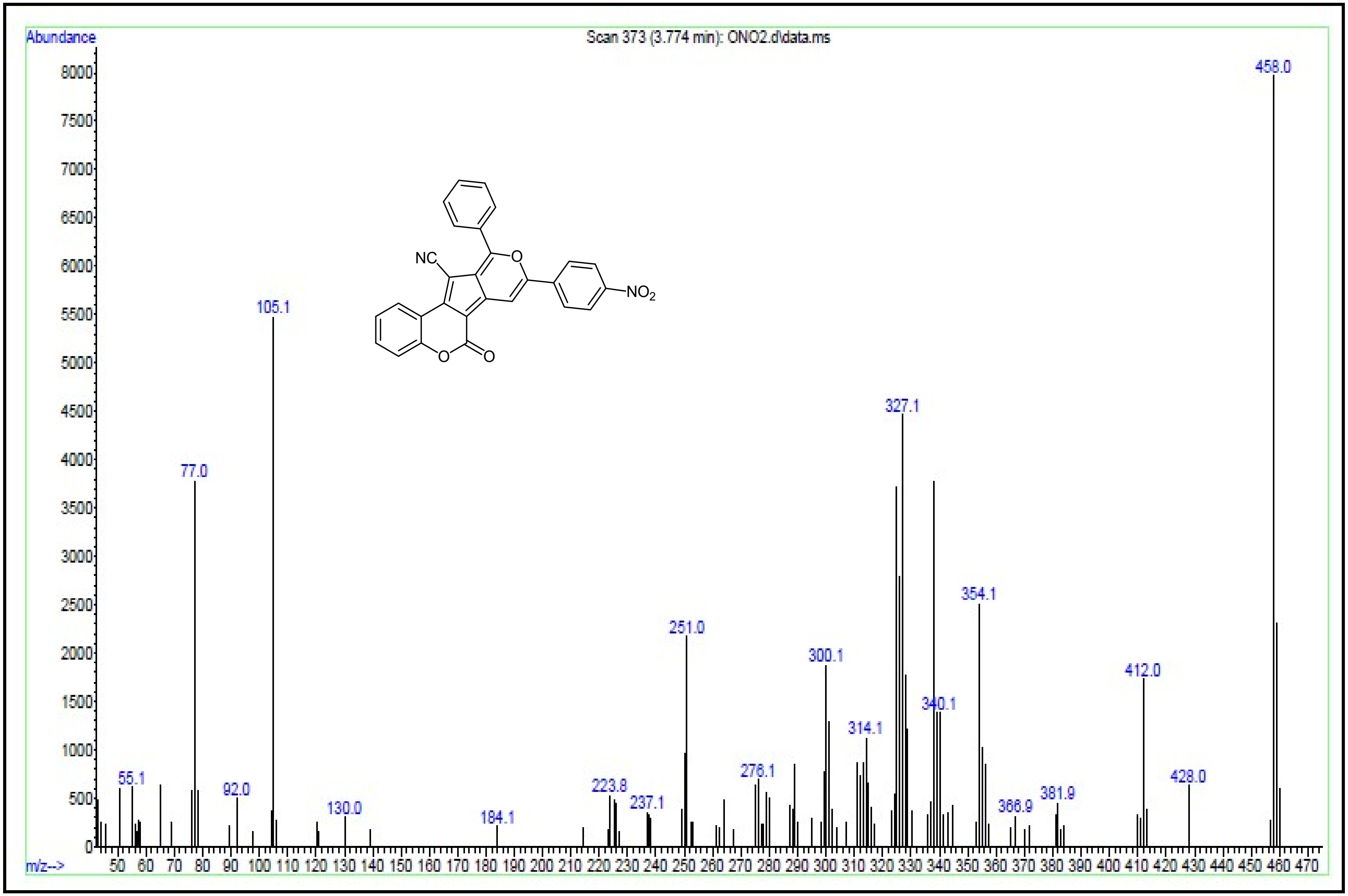

Mass spectrum of **3f**

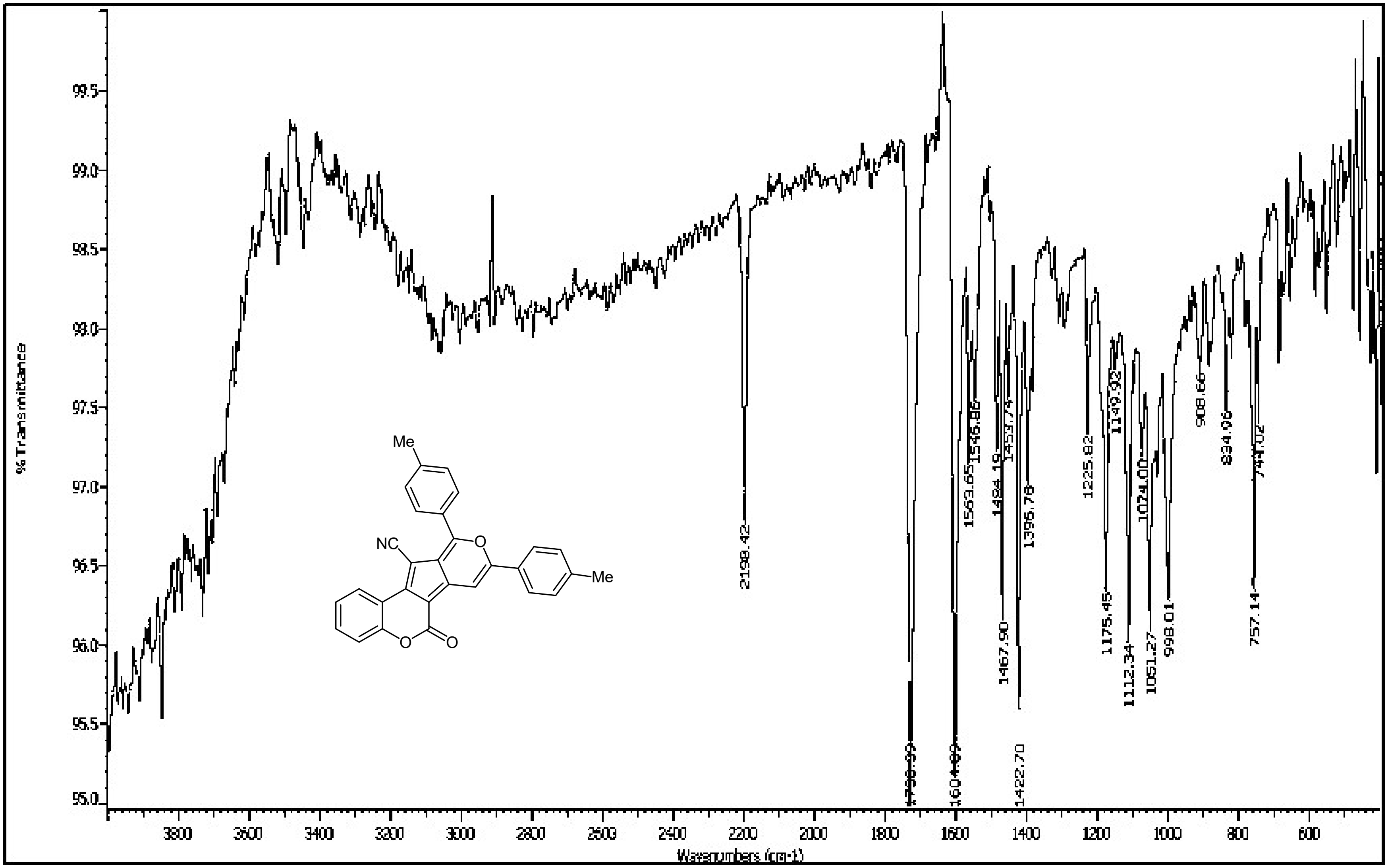

IR spectrum of **3g**

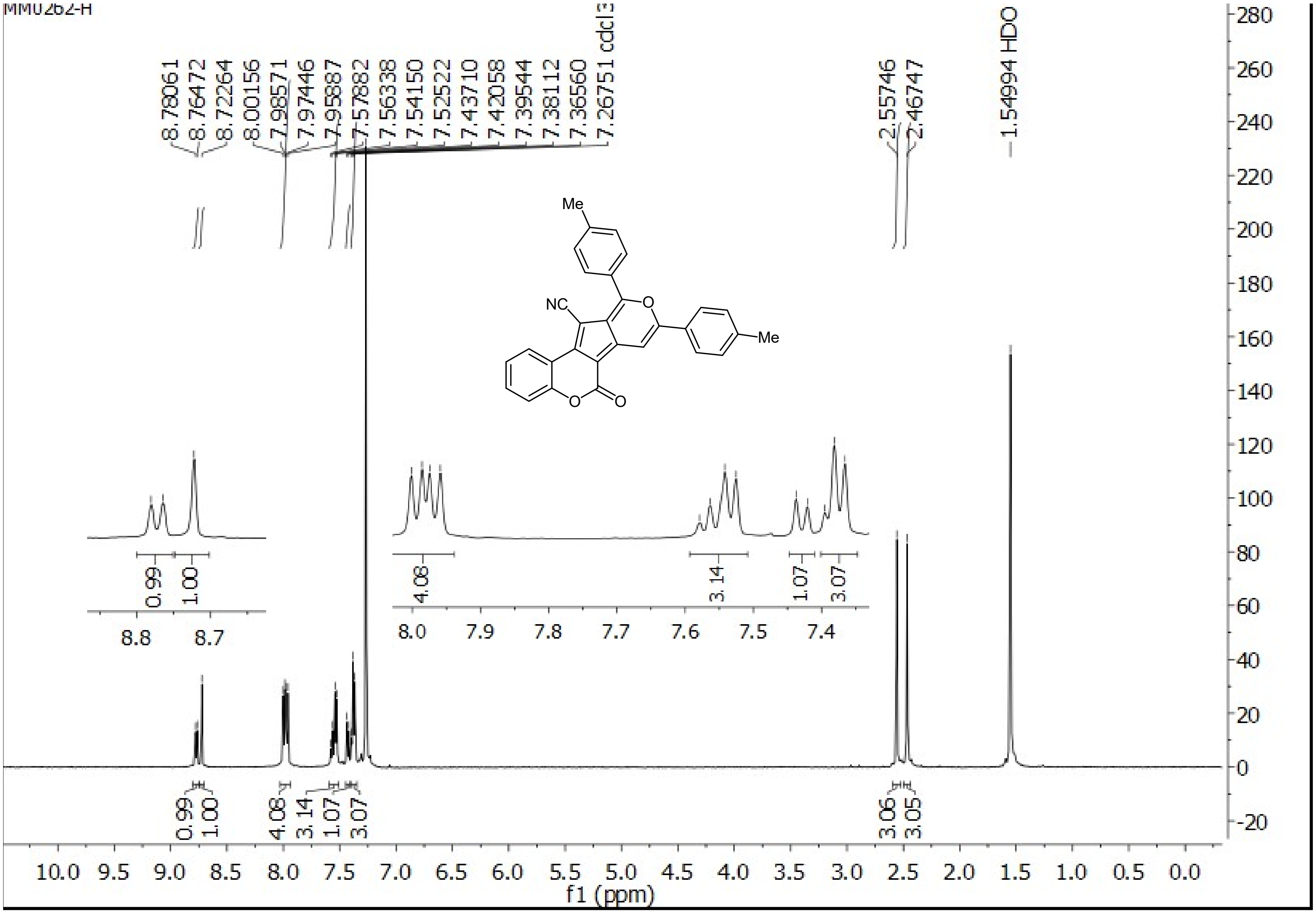

^{1}H NMR ($CDCl_3$, 500 MHz) of **3g**

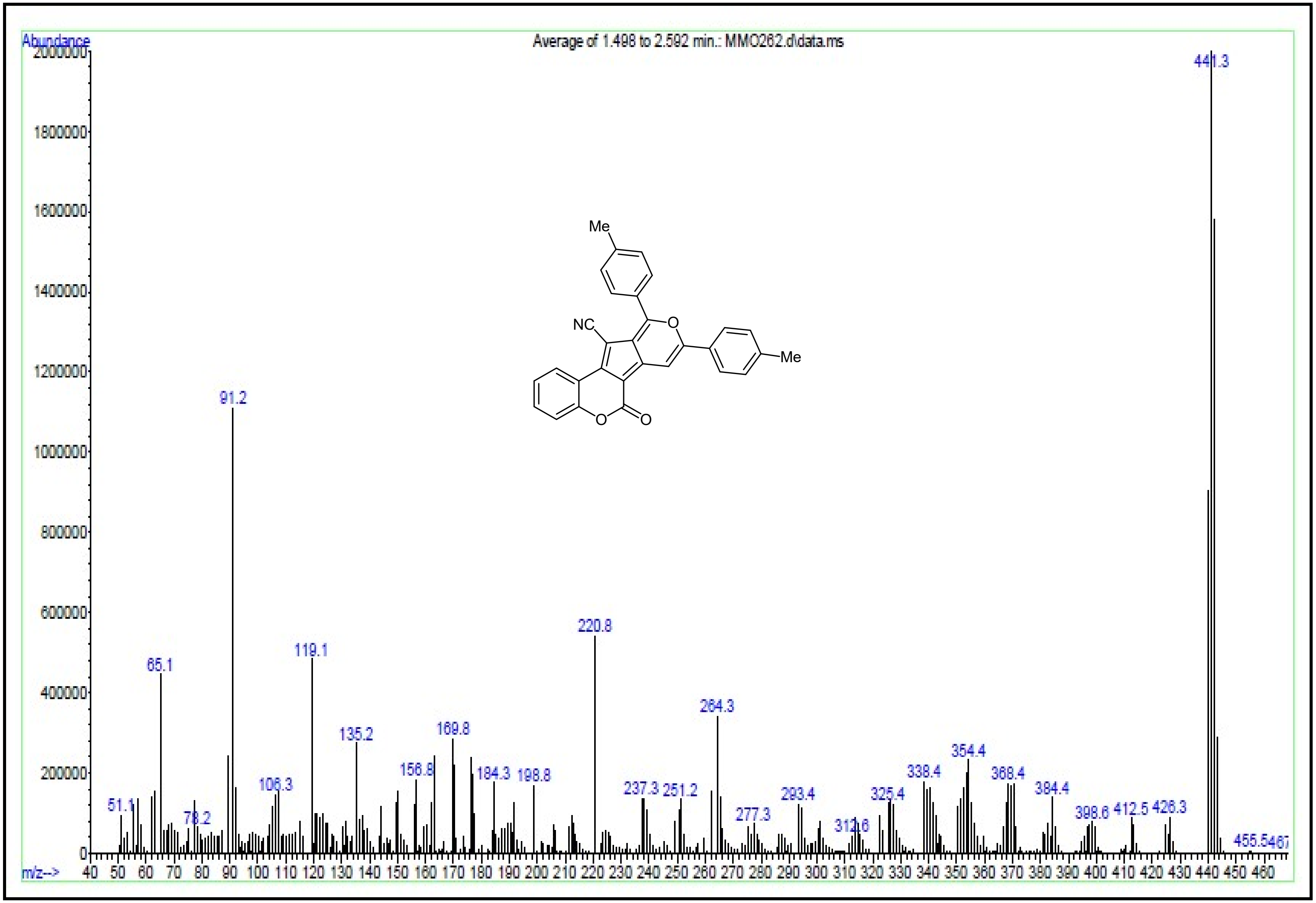

Mass spectrum of **3g**

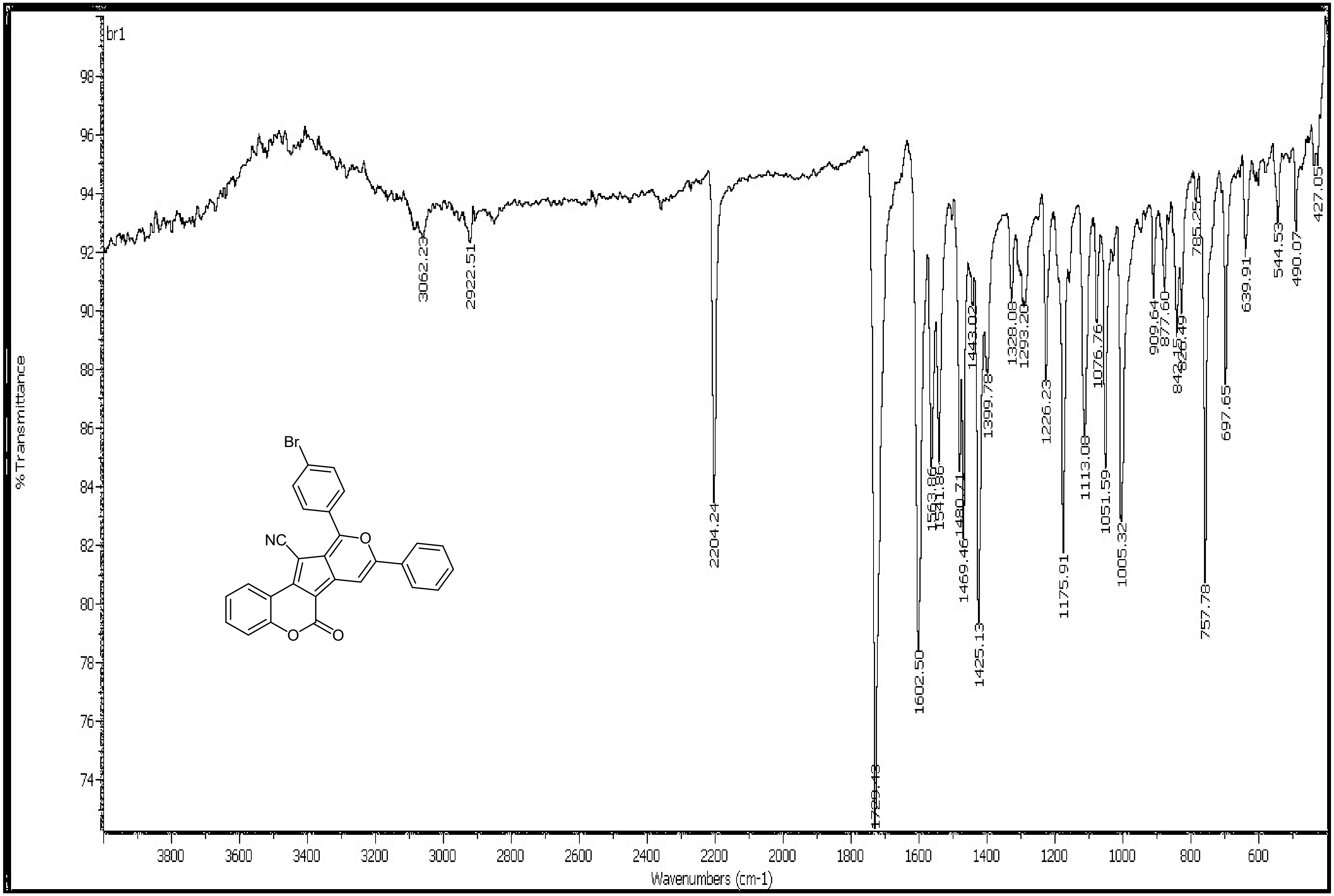

IR spectrum of **3h**

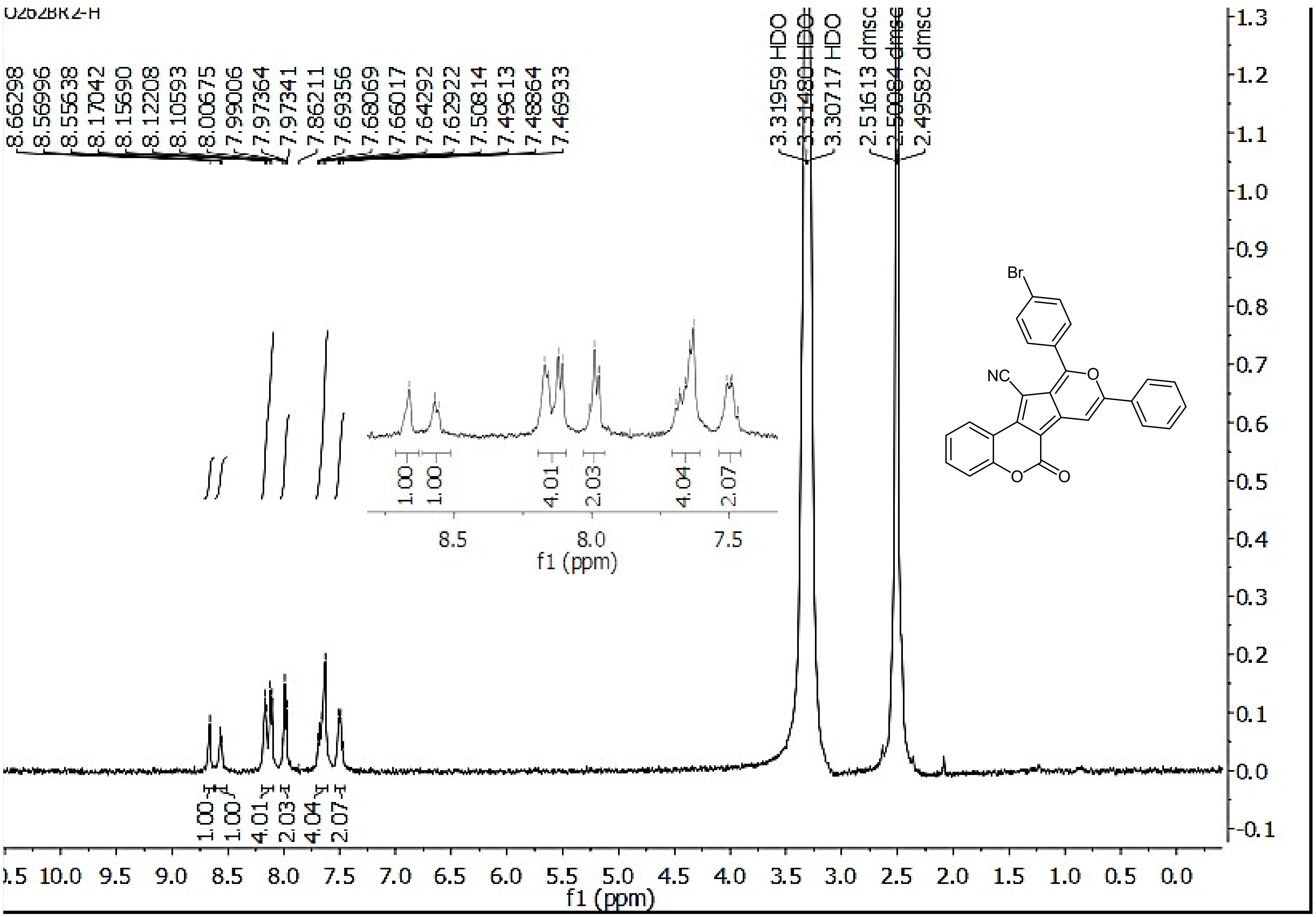

^{1}H NMR (DMSO-d_6, 500 MHz) of **3h**

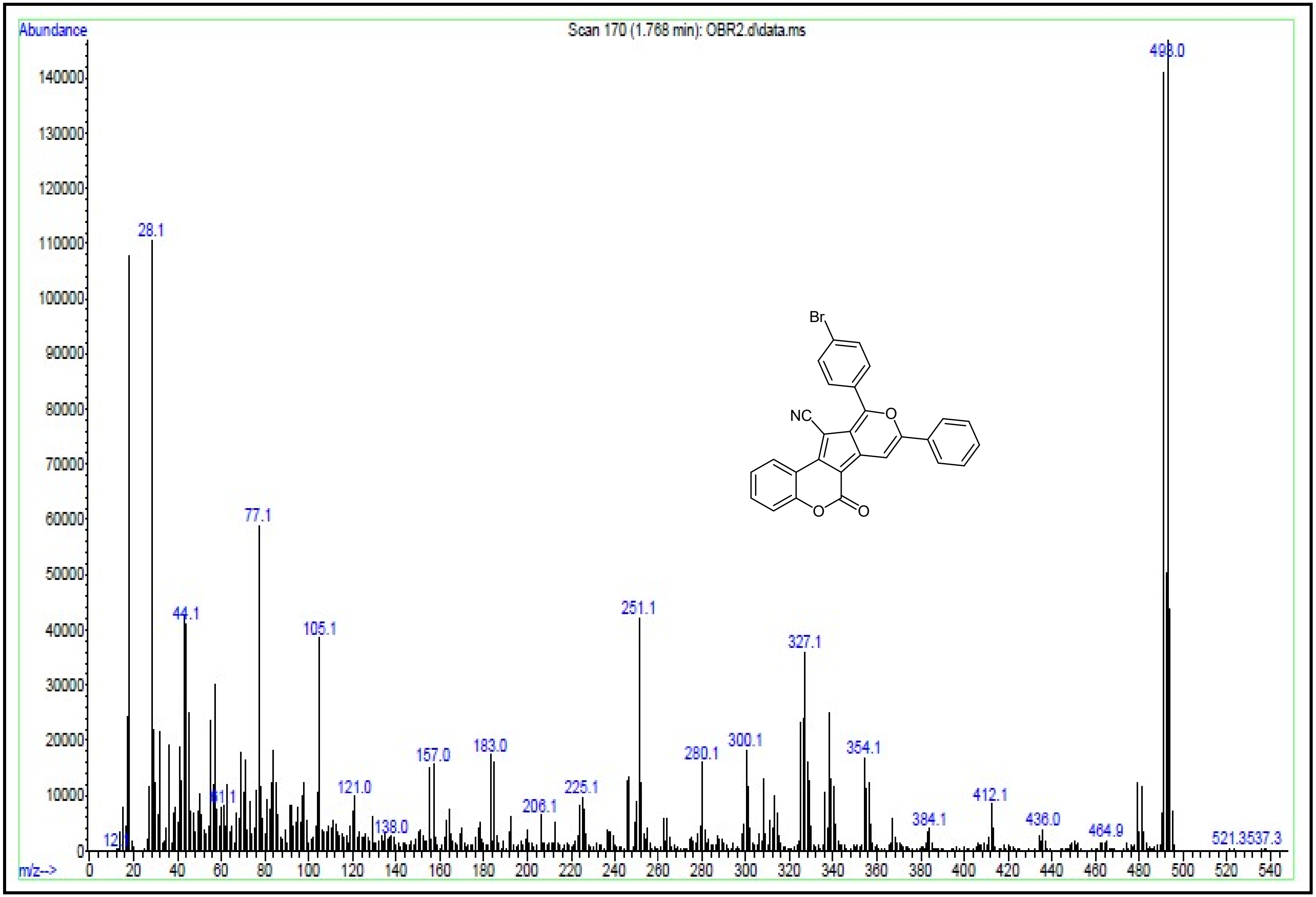

Mass spectrum of **3h**

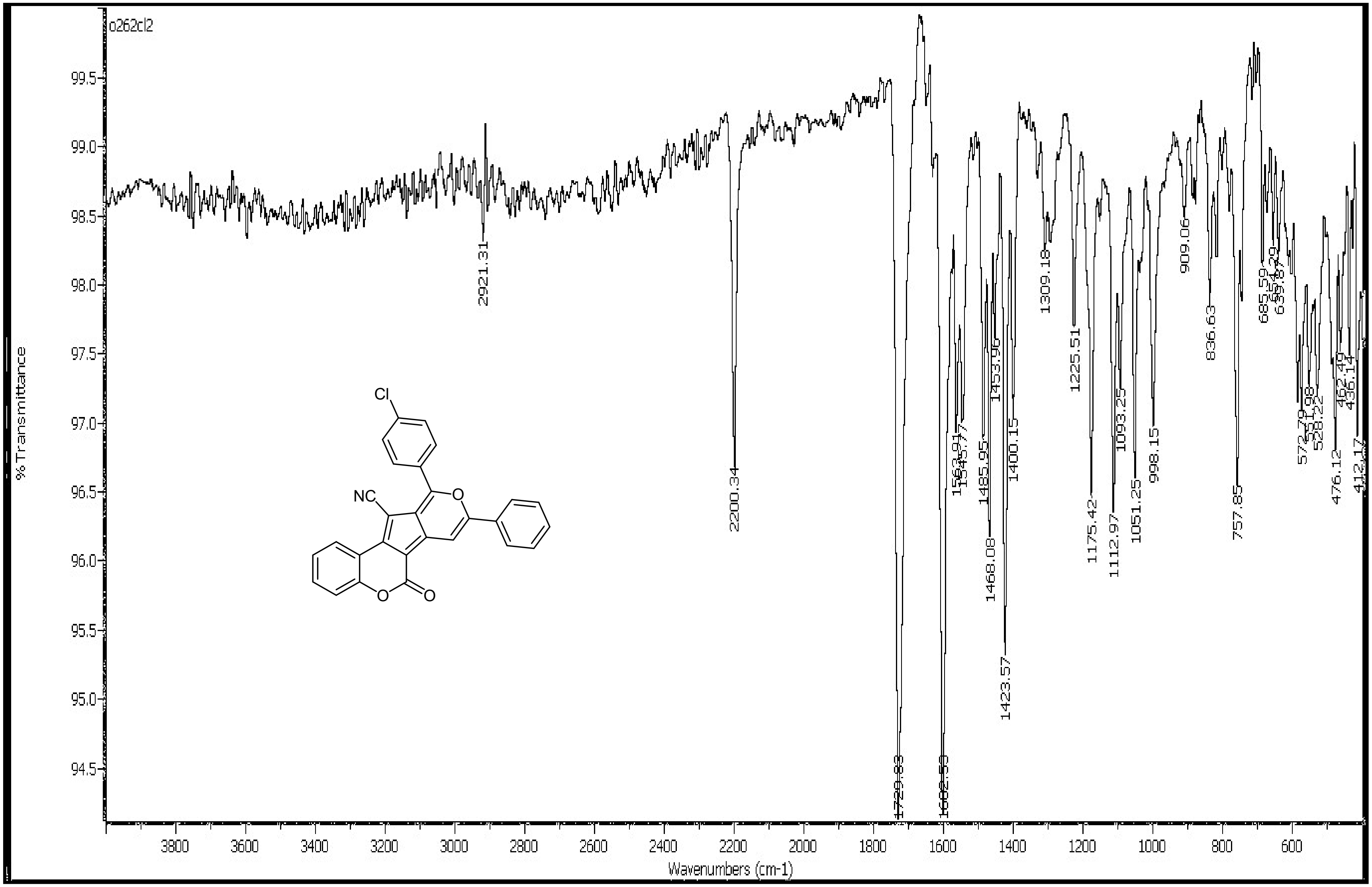

IR spectrum of **3i**

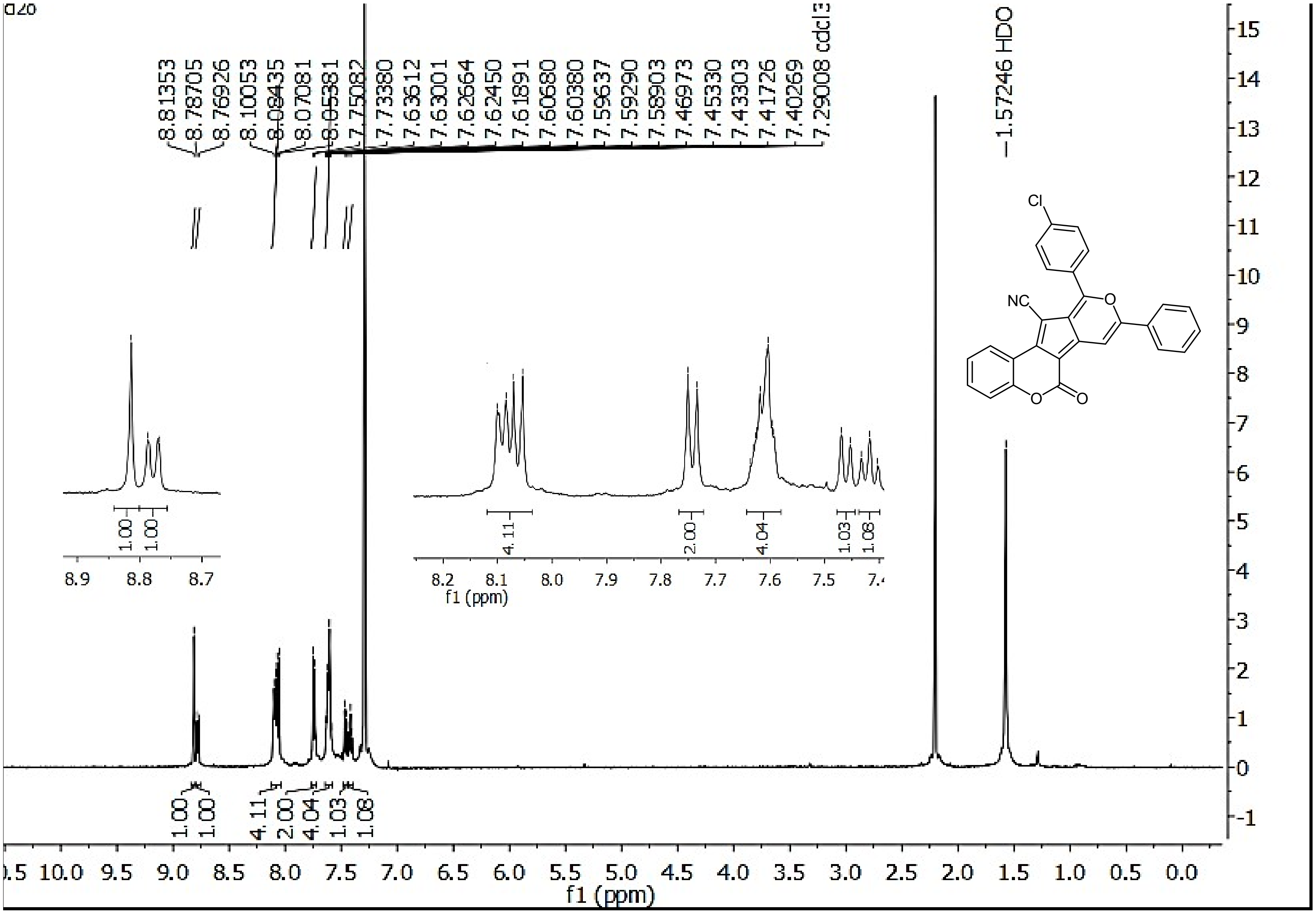

^1H NMR ($CDCl_3$, 500 MHz) of **3i**

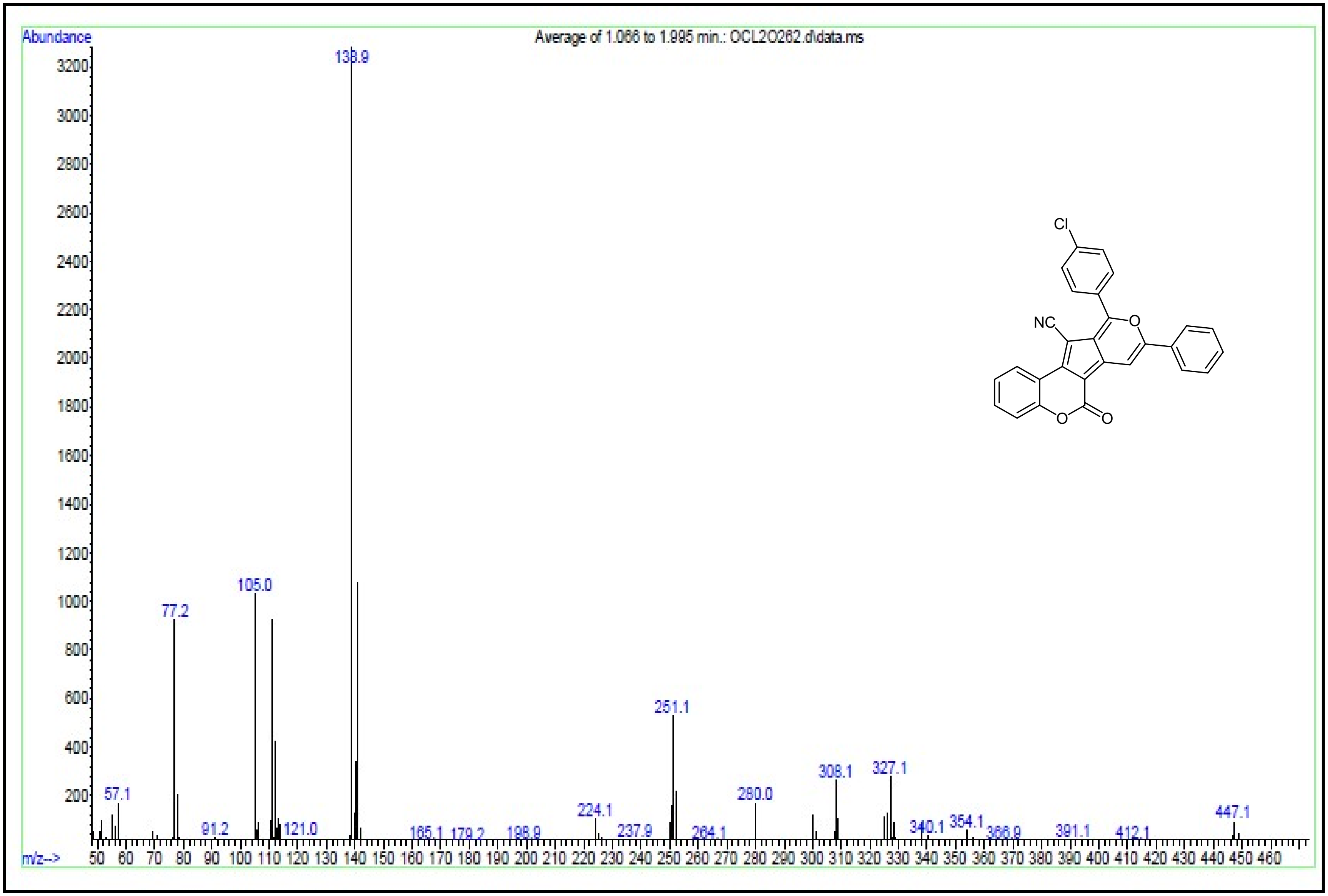

Mass spectrum of **3i**

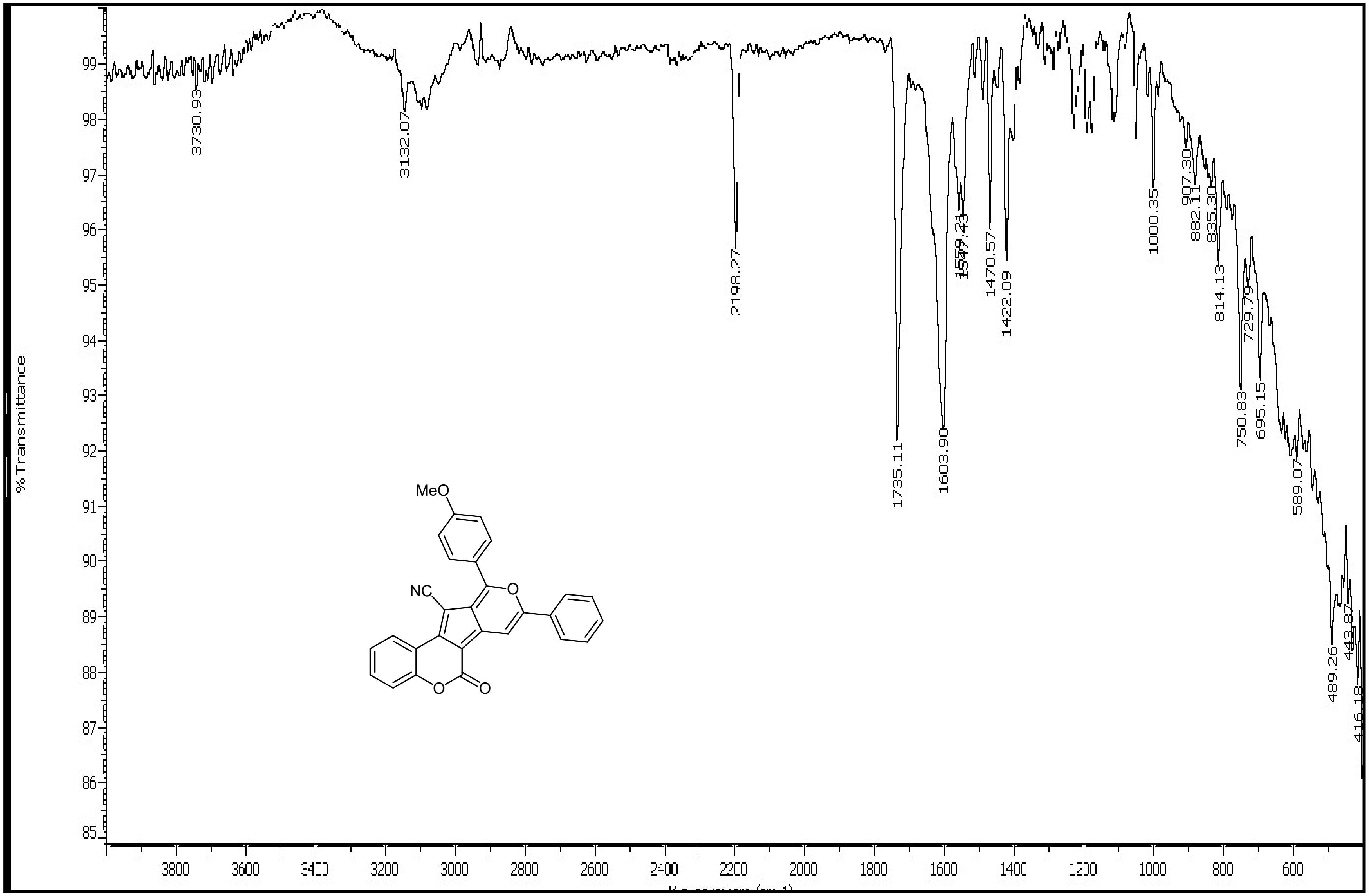

IR spectrum of **3j**

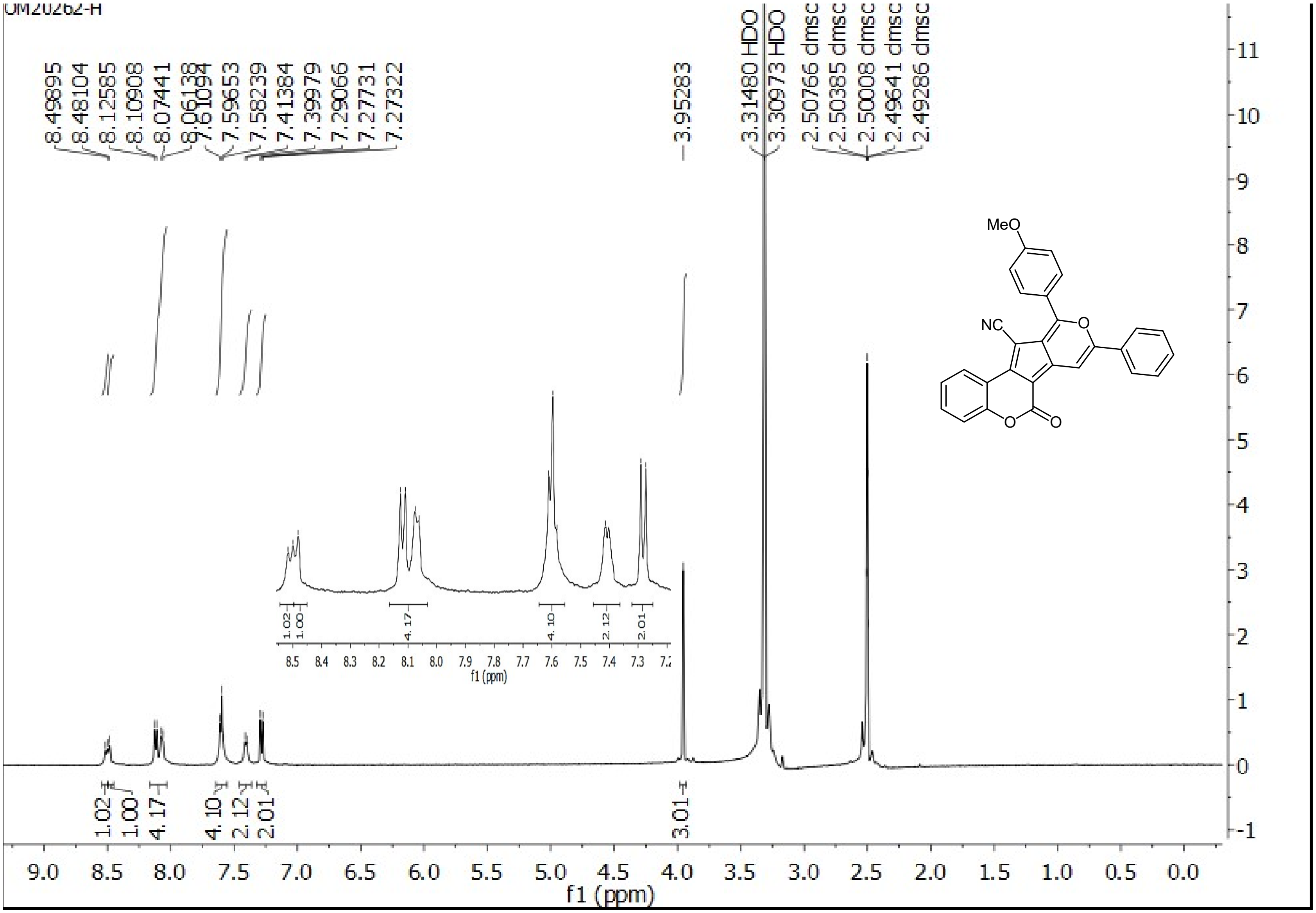

^{1}H NMR (DMSO-d_6, 500 MHz) of **3j**

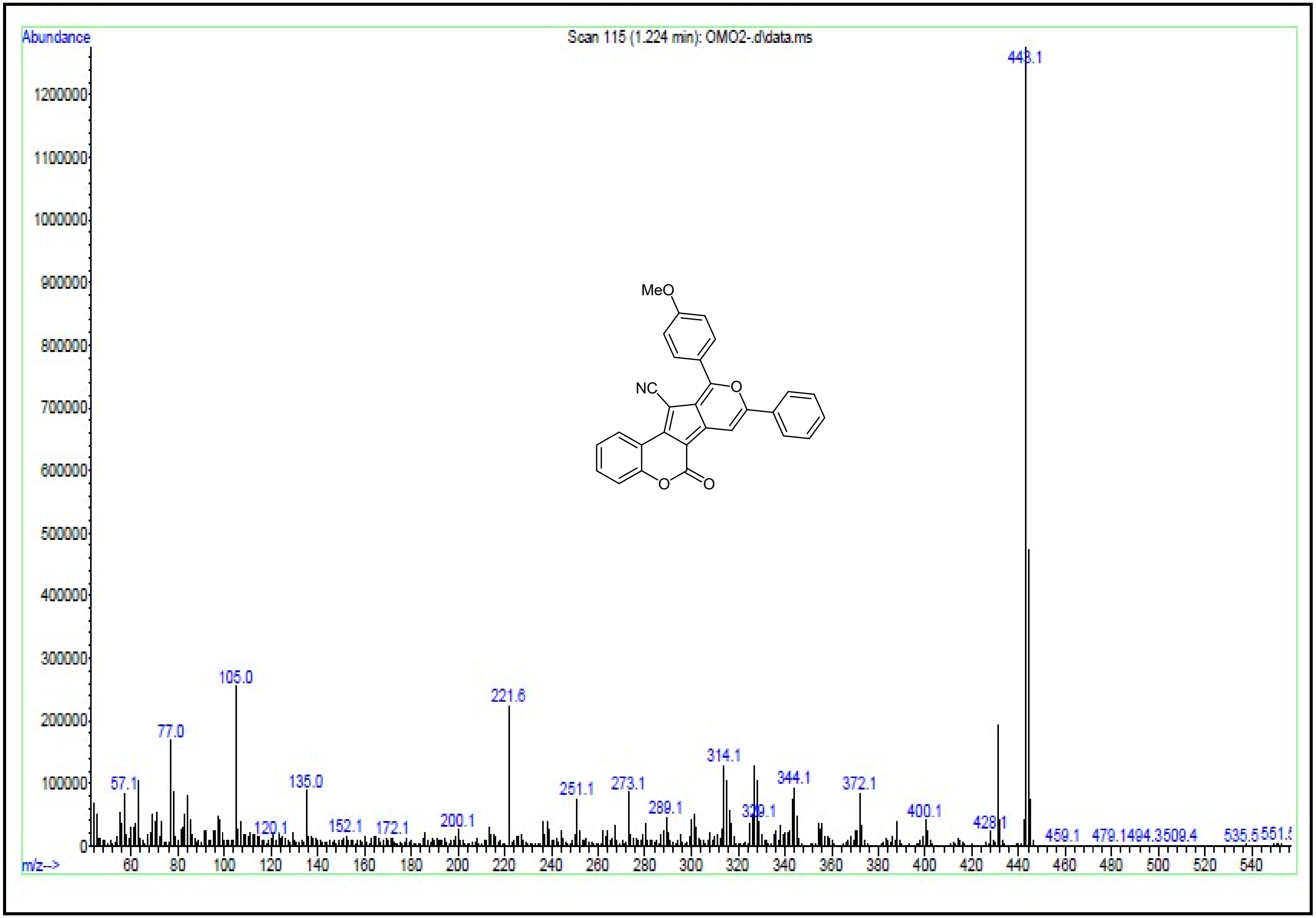

Mass spectrum of **3j**

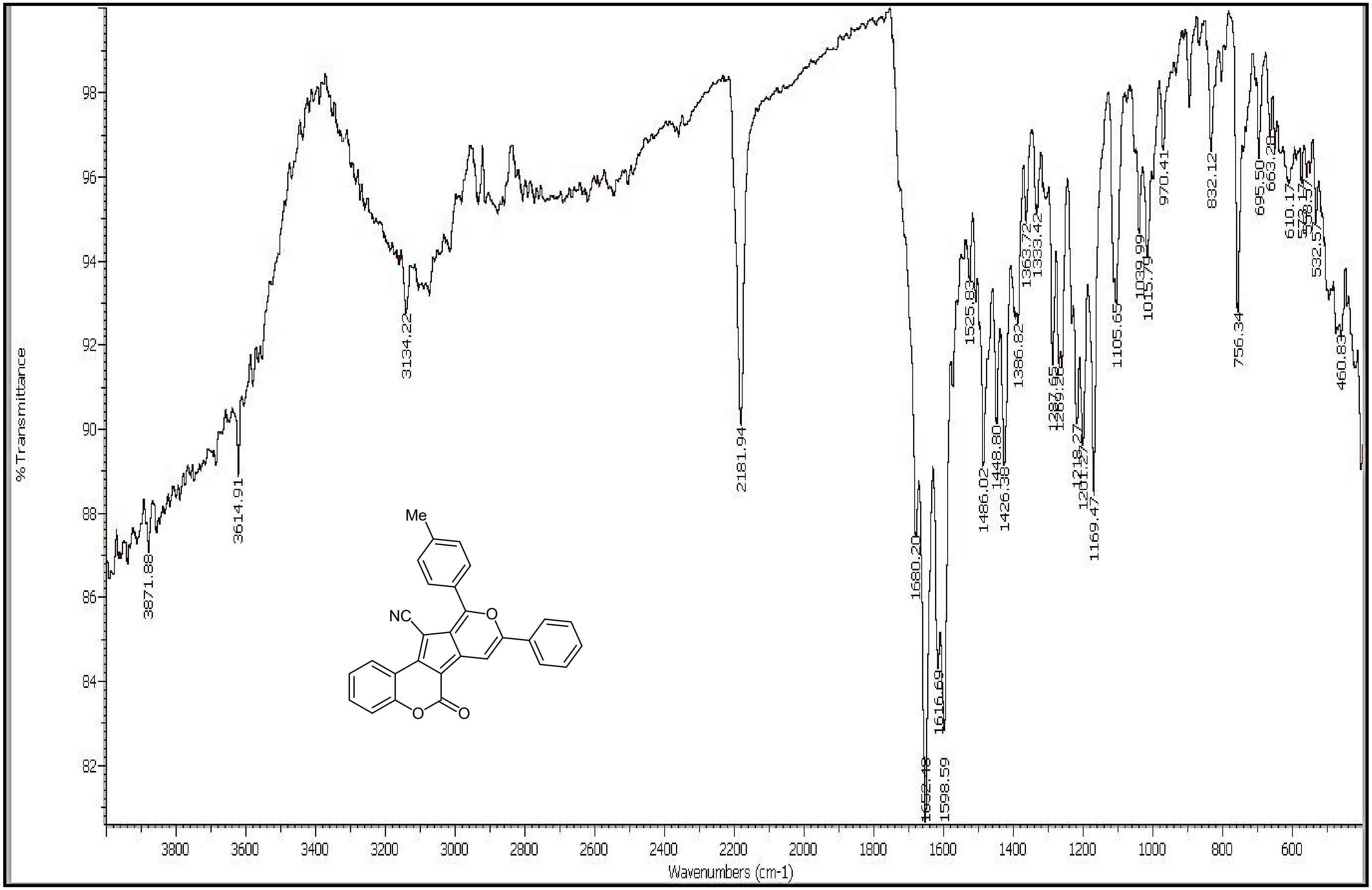

IR spectrum of **3k**

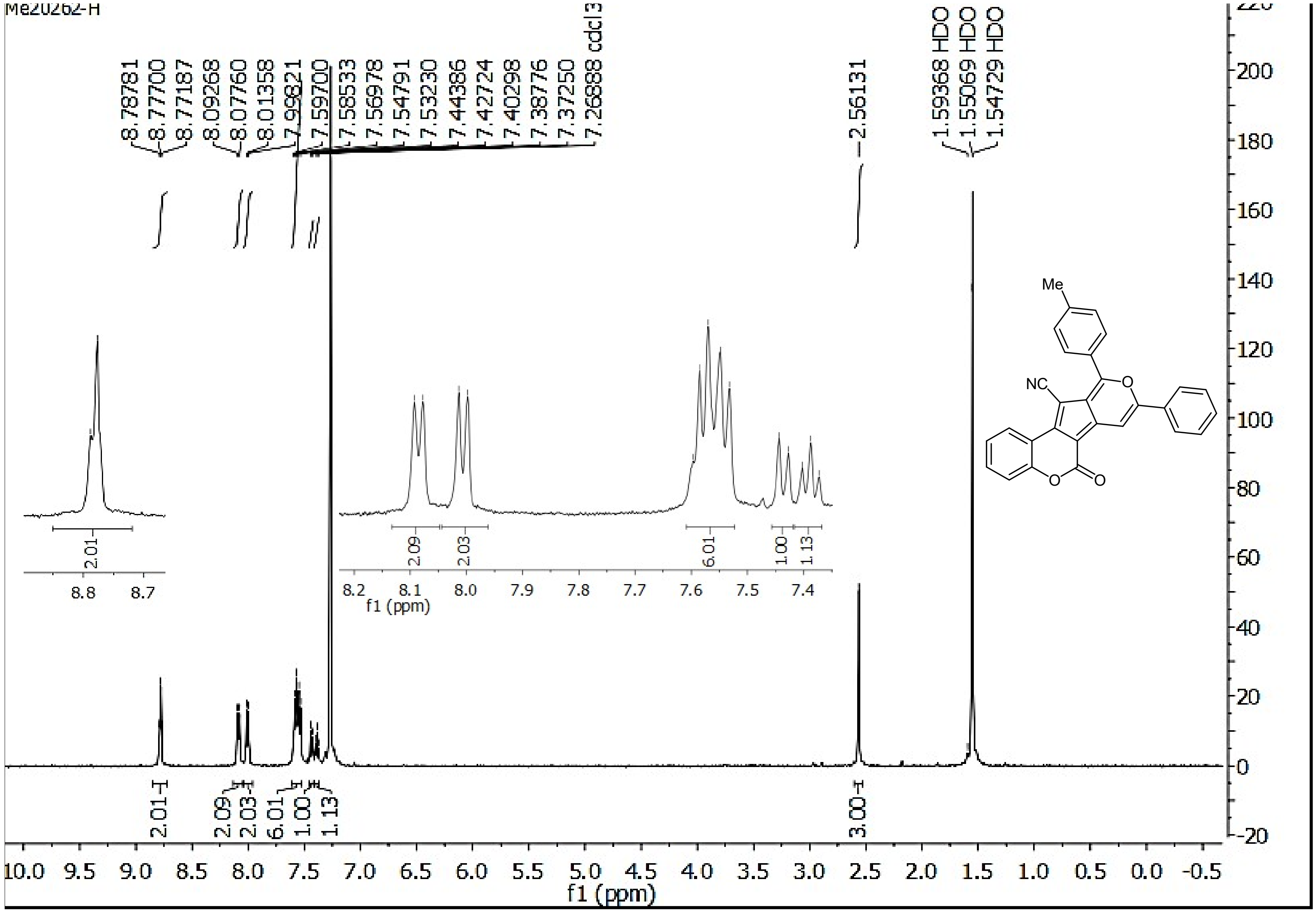

^{1}H NMR ($CDCl_3$, 500 MHz) of **3k**

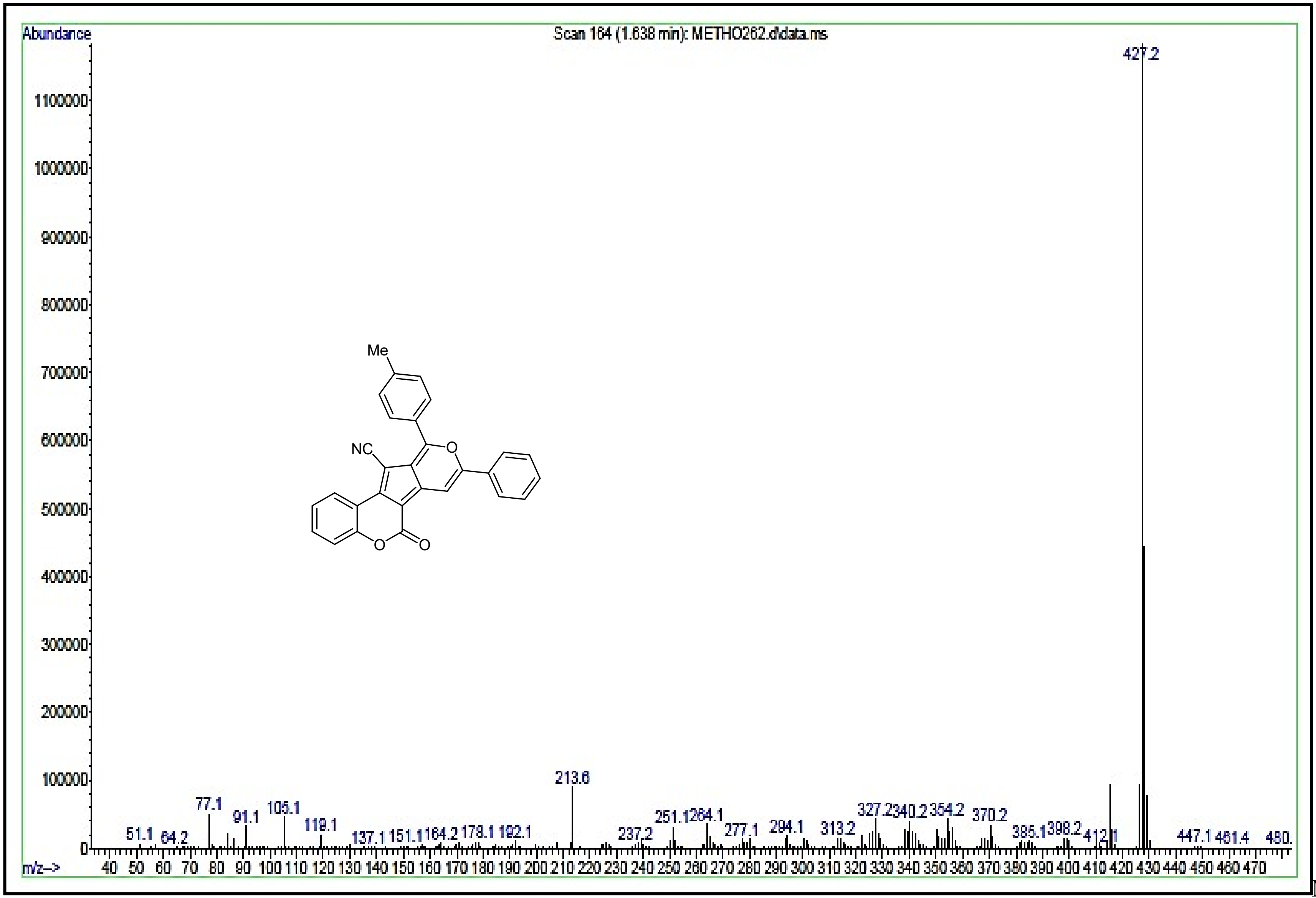

Mass spectrum of **3k**
